# Supplementary material for: Global mapping and evolution of persistent fronts in Large Marine Ecosystems over the past 40 years
Source: Nat Commun. 2024 May 14;15:4090. doi: 10.1038/s41467-024-48566-w (PMC11094120; doi:10.1038/s41467-024-48566-w)
Supplement: Supplementary file 1 — Supplementary Information [file 41467_2024_48566_MOESM1_ESM.pdf]

Supplementary information for

Global mapping and evolution of persistent fronts in Large Marine Ecosystems over the past 40 years

Qinwang Xing[1], Haiqing Yu[1*], Hui Wang[1,2,3]

*Corresponding author. Email: yuhaiqing@sdu.edu.cn

This file includes:

- Supplementary Figs. 1 to 11
- Supplementary Table 1

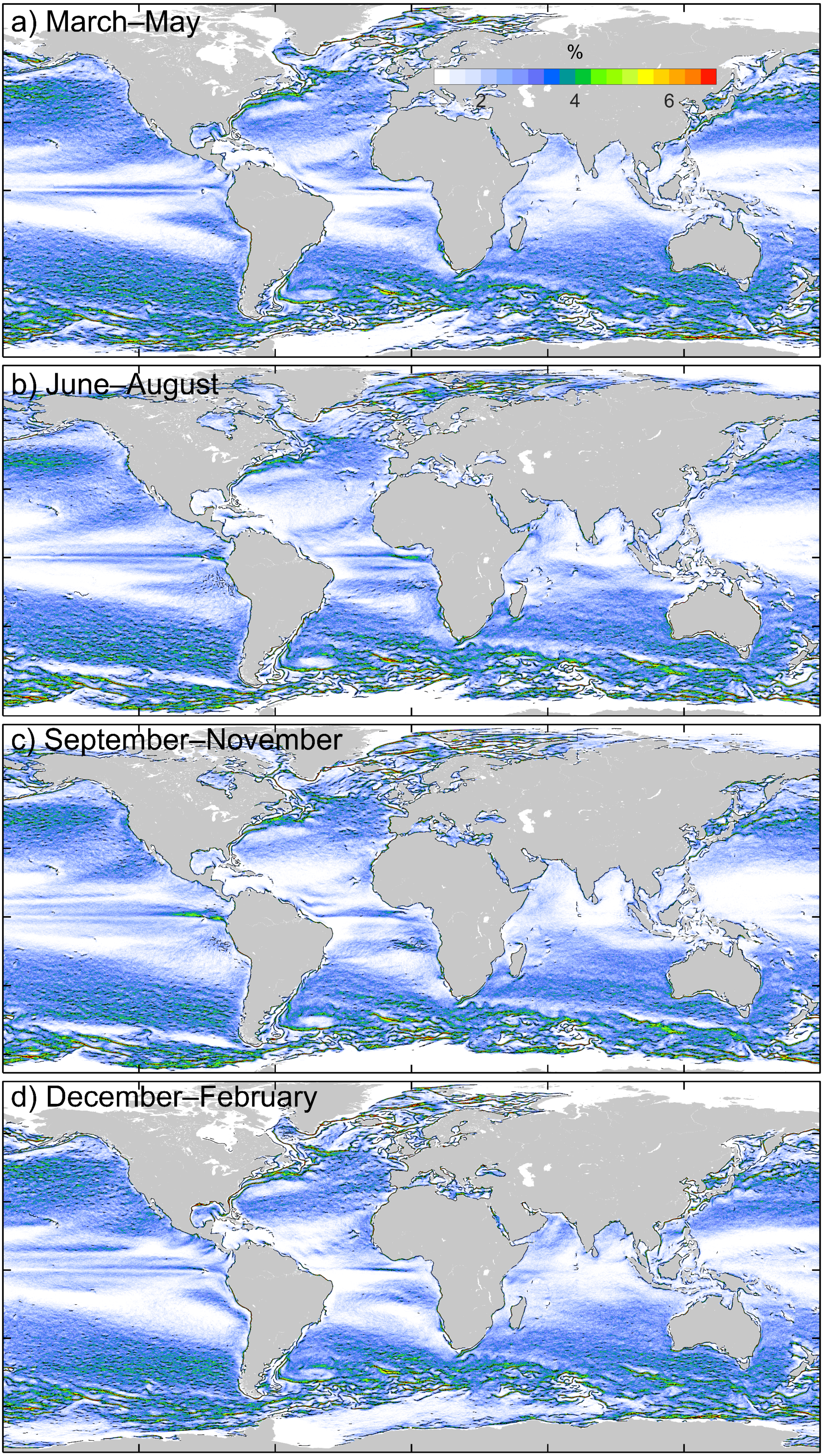
a) March–May
%
2
4
6
b) June–August
c) September–November
d) December–February

Supplementary Fig. 1 | Seasonal frontal occurrence fields from 1982 to 2021. **a**, **b**, **c**, **d**, correspond to the four seasons. Black lines represent the identified persistent frontal segments.

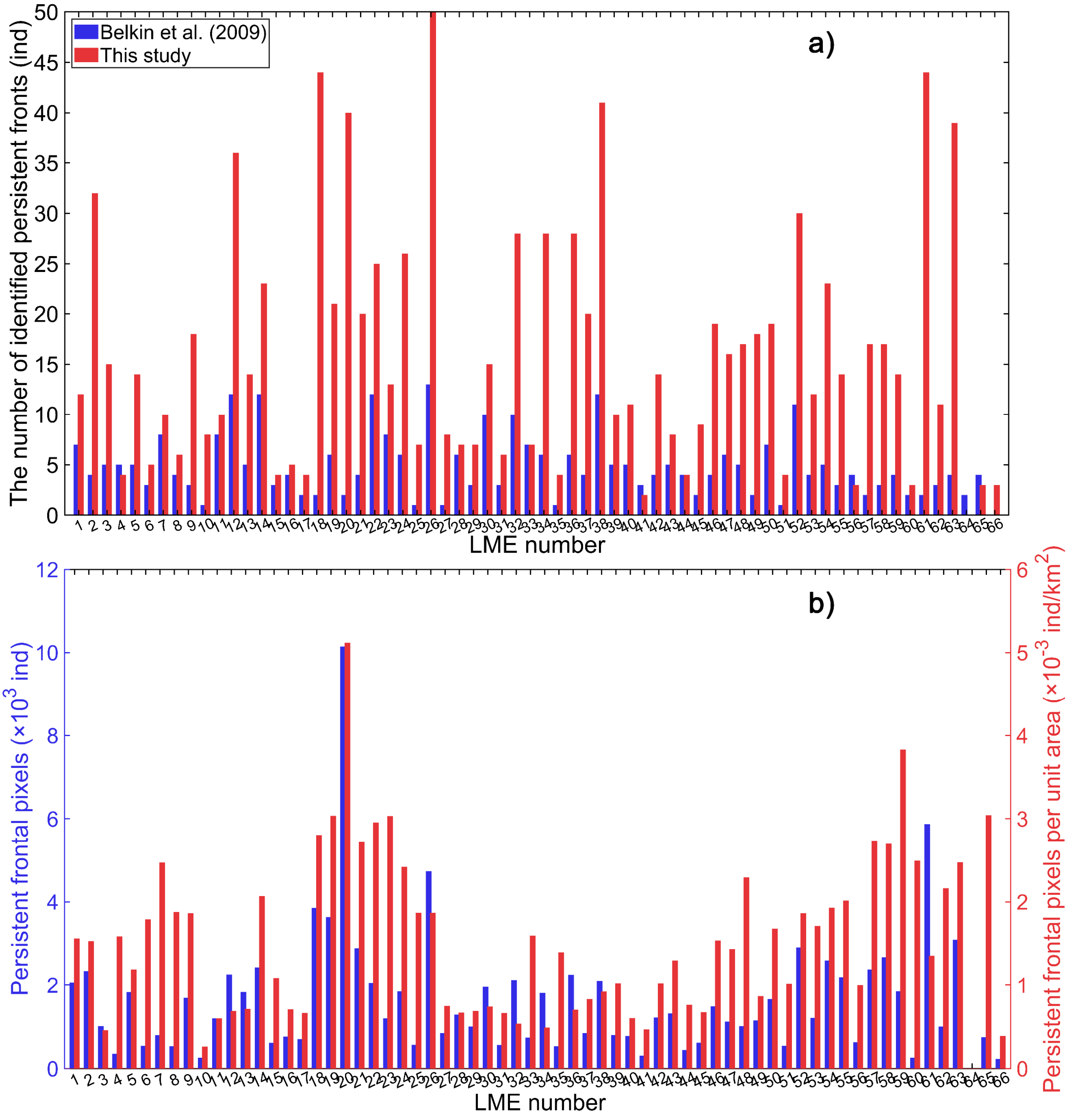

Supplementary Fig. 2 | The number of identified persistent fronts in each Large Marine Ecosystem (LME). **a**, Comparison of the number of identified persistent fronts between the results of Belkin et al. (2009)[1] and this study. **b**, The number of identified persistent frontal pixels in each LME.

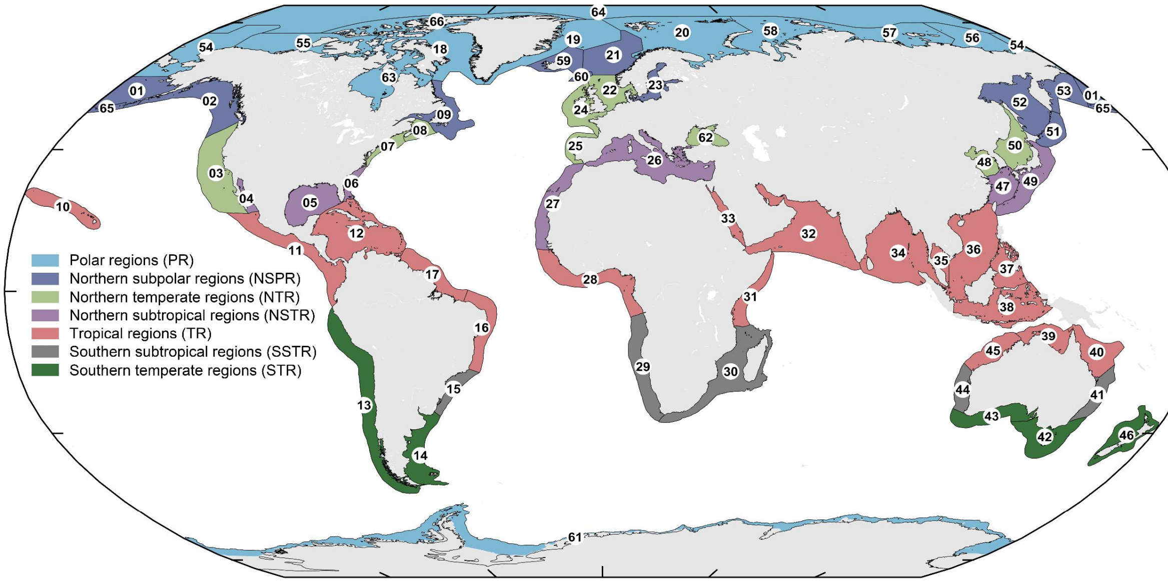

01 East Bering Sea
02 Gulf of Alaska
03 California Current
04 Gulf of California
05 Gulf of Mexico
06 Southeast U.S. Continental Shelf
07 Northeast U.S. Continental Shelf
08 Scotian Shelf
09 Labrador-Newfoundland
10 Insular Pacific-Hawaiian
11 Pacific Central -American Coastal
12 Caribbean Sea
13 Humboldt Current
14 Patagonian Shelf
15 South Brazil Shelf
16 East Brazil Shelf
17 North Brazil Shelf
18 Canadian Eastern Arctic -West Greenland
19 Greenland Sea
20 Barents Sea
21 Norwegian Sea
22 North Sea
23 Baltic Sea
24 Celtic-Biscay Shelf
25 Iberian Coastal
26 Mediterranean Sea
27 Canary Current
28 Guinea Current
29 Benguela Current
30 Agulhas Current
31 Somali Coastal Current
32 Arabian Sea
33 Red Sea
34 Bay of Bengal
35 Gulf of Thailand
36 South China Sea
37 Sulu-Celebes Sea
38 Indonesian Sea
39 North Australian Shelf
40 Northeast Australian Shelf
41 East Central Australian Shelf
42 Southeast Australian Shelf
43 South West Australian Shelf
44 West Central Australian Shelf
45 Northwest Australian Shelf
46 New Zealand Shelf
47 East China Sea
48 Yellow Sea
49 Kuroshio Current
50 Sea of Japan
51 Oyashio Current
52 Sea of Okhotsk
53 West Bering Sea
54 Northern Bering-Chukchi Seas
55 Beaufort Sea
56 East Siberian Sea
57 Laptev Sea
58 Kara Sea
59 Iceland Shelf and Sea
60 Faroe Plateau
61 Antarctica
62 Black Sea
63 Hudson Bay Complex
64 Central Arctic
65 Aleutian Islands
66 Canadian High Arctic -North Greenland

Supplementary Fig. 3 | Large Marine Ecosystems (LMEs) distribution. Different colors correspond to various latitudinal regions, and the overlapping numbers within each LME represent the LME numbers.

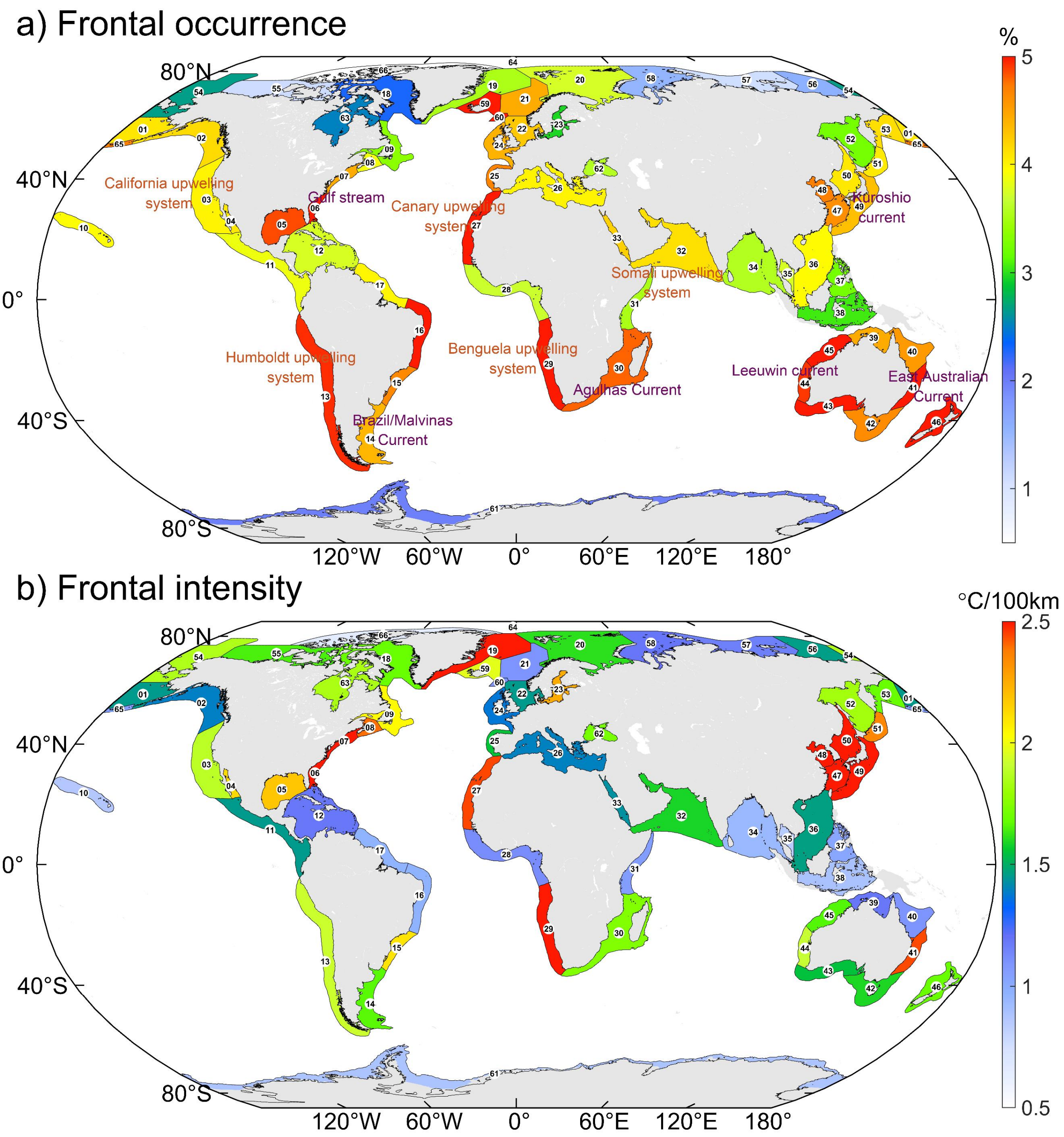

Supplementary Fig. 4 | Large Marine Ecosystem (LME)-integrated persistent fronts from 1982 to 2021. **a**, **b**, integrated occurrence (**a**) and intensity (**b**) of persistent fronts within each LME. The overlapping numbers within each LME represent the LME numbers.

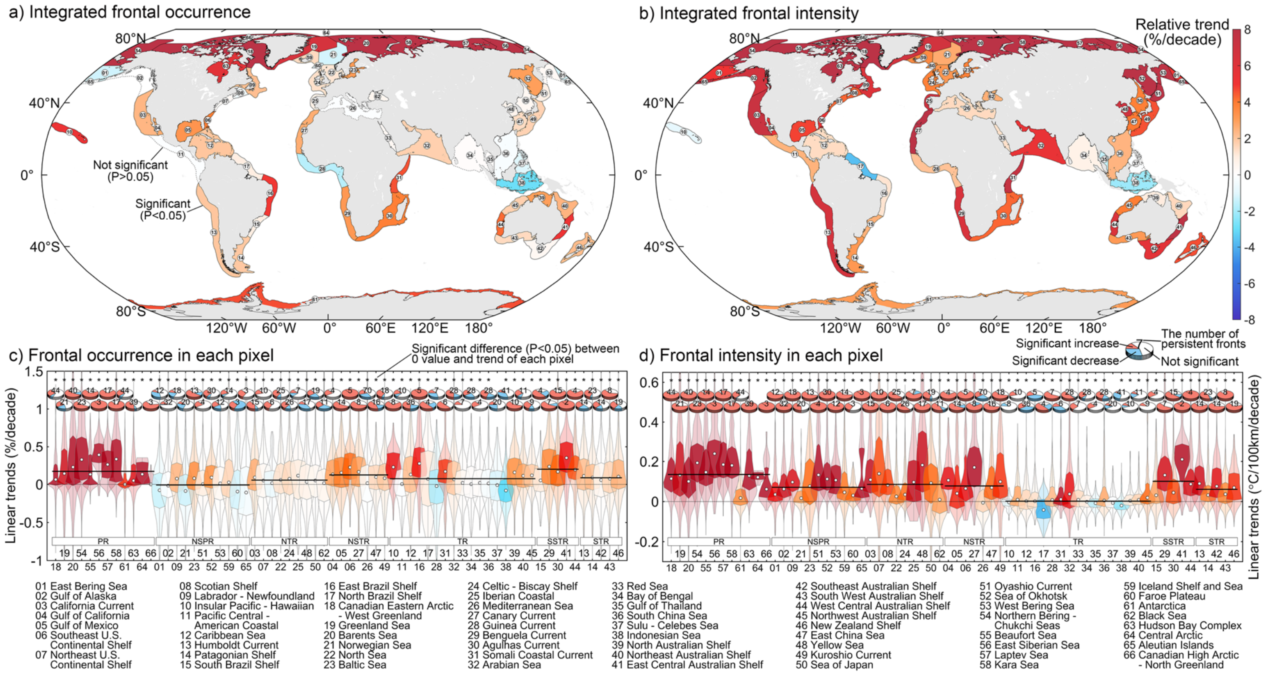

Supplementary Fig. 5 | Long-term changes in persistent fronts from 1982 to 2021. **a**, **b**, Large Marine Ecosystem (LME)-integrated relative trends in occurrence (**a**) and intensity (**b**) of persistent fronts. Solid boundary lines of the LMEs denote statistically significant trends (*P*<0.05). The overlapping numbers within each LME represent the LME numbers. **c**, **d**, Trends in occurrence (**c**) and intensity (**d**) of each persistent frontal pixel. The shadows of violin plots represent the middle 50% of the trends, with hollow points denoting the medians, and asterisks indicated trends that are significantly greater or less than 0 in each LME. The horizontal lines represent the average trends of each pixels in different regions, while pie charts illustrate the proportion of persistent fronts with increasing, decreasing, and non-significant trends. The numbers within the pie charts indicate the total quantity of persistent fronts in each LME.

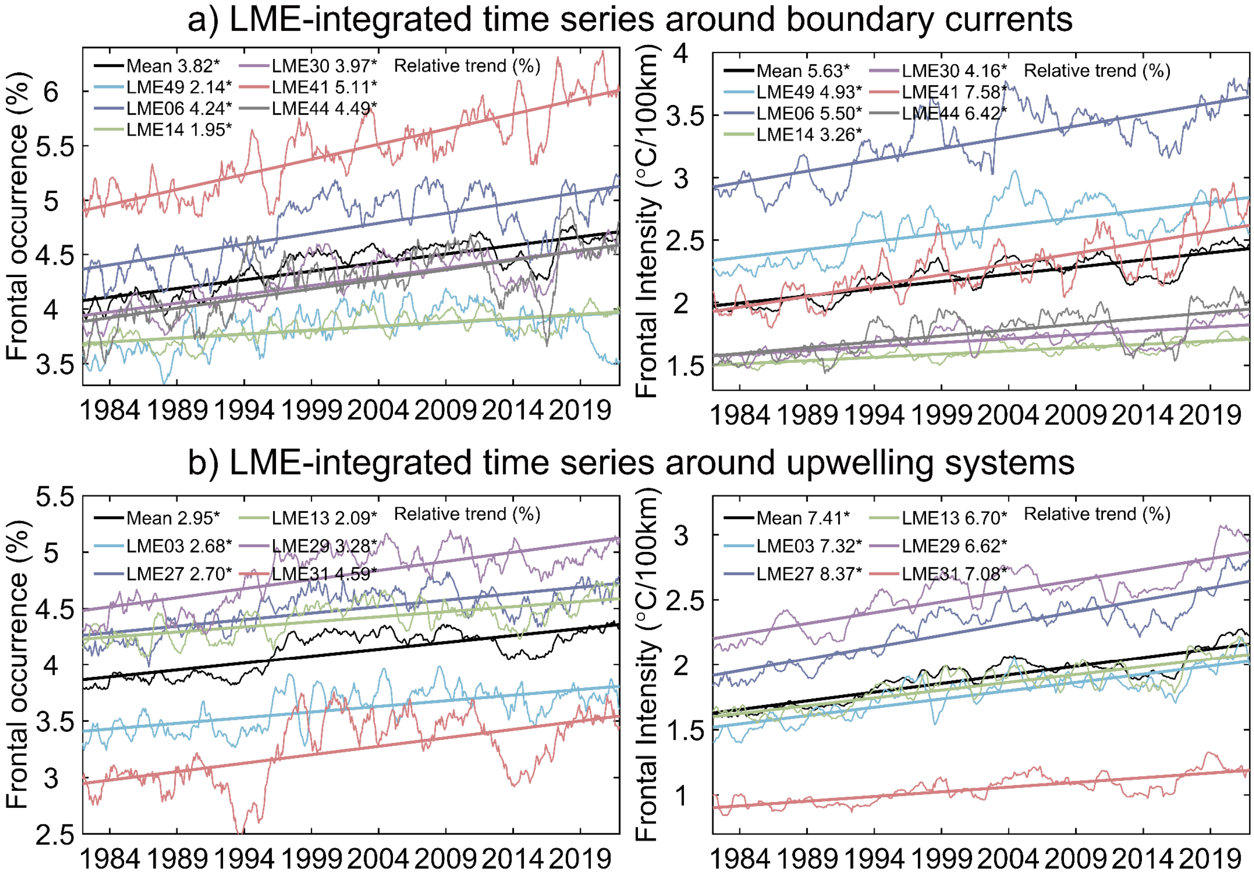

Supplementary Fig. 6 | Large Marine Ecosystem (LME)-integrated long-term changes in persistent fronts. **a**, **b**, The time series of frontal occurrence and intensity around the boundary current (**a**), upwelling system (**b**). The number represents the relative trend of long-term changes in persistent fronts within each LME, with asterisks denoting significance at the 0.05 level.

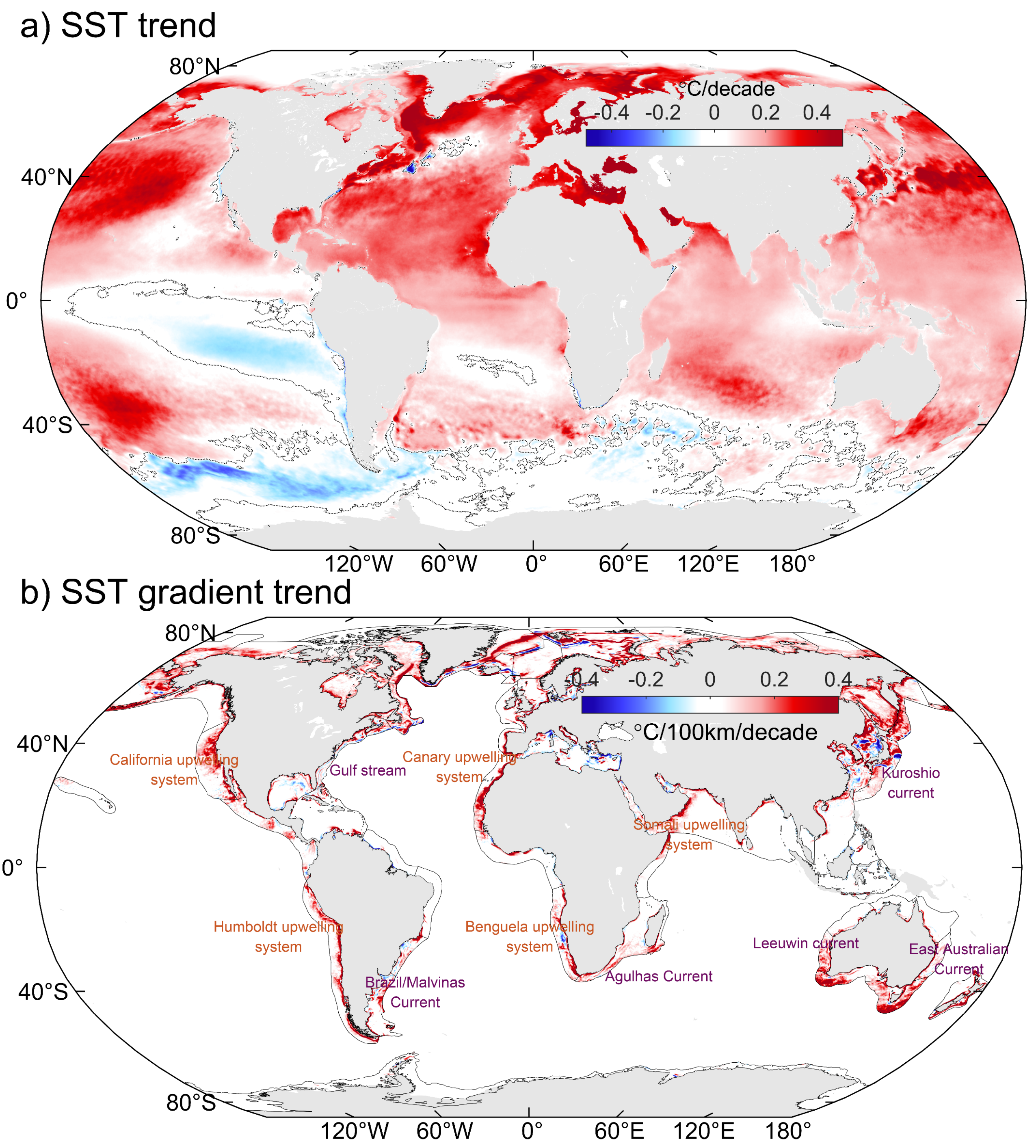

Supplementary Fig. 7 | Long-term changes in SST and SST gradient. **a**, linear trends of SST during 1982 to 2021. The dotted lines represent the zero contour lines of the trends. **b**, linear trends of SST gradient during 1982 to 2021.

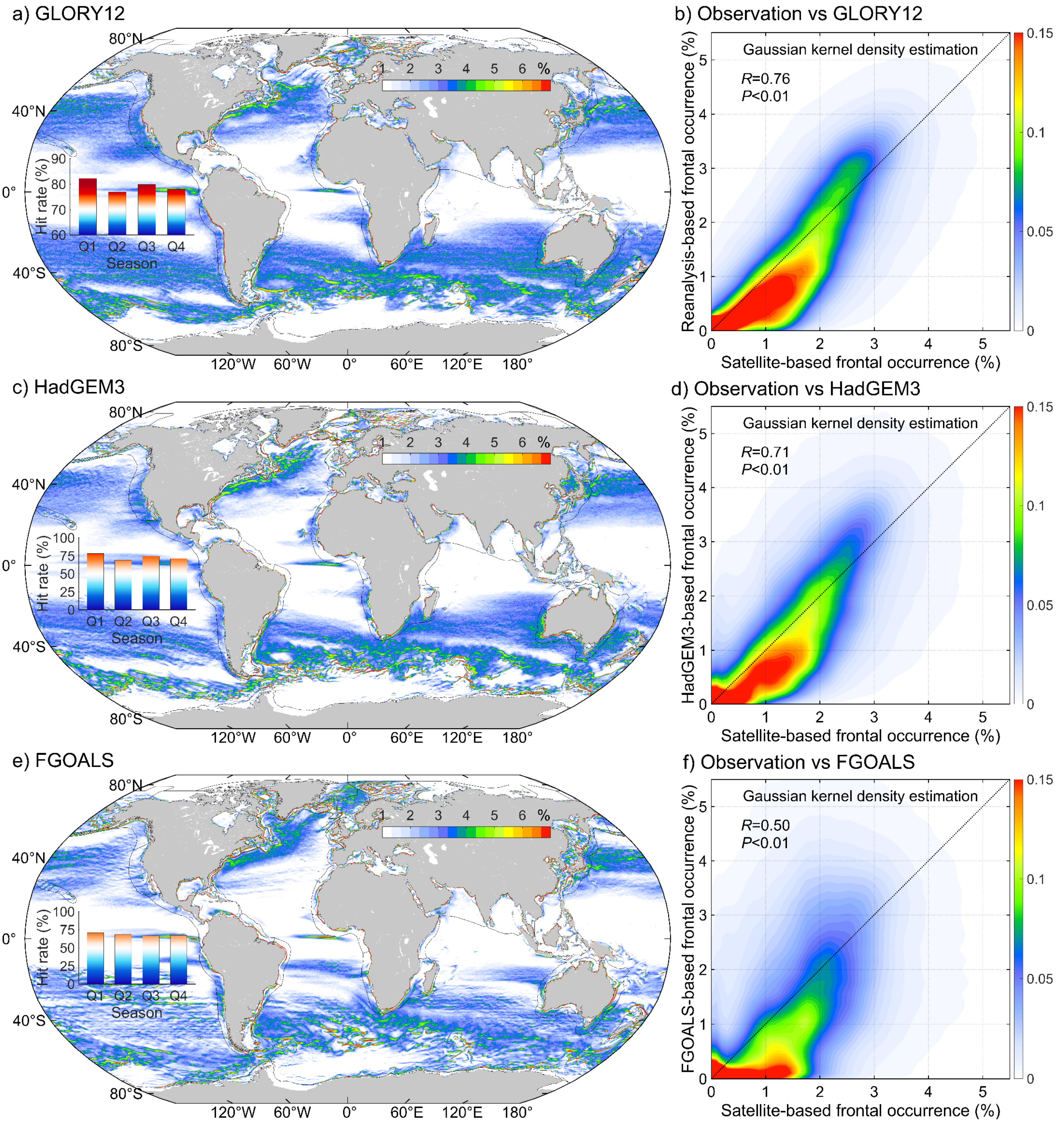

Supplementary Fig. 8 | Comparisons of the frontal occurrence between satellite observation and analysis data or climate models within LMEs. **a**, **c**, **e**, the frontal occurrence fields from analysis data GLORY12 (**a**) as well as climate models HadGEM3 (**c**) and FGOALS (**e**). **b**, **d**, **f**, comparisons of the frontal occurrence fields between satellite observation and analysis data GLORY12 (**b**) as well as climate models HadGEM3 (**d**) and FGOALS (**f**).

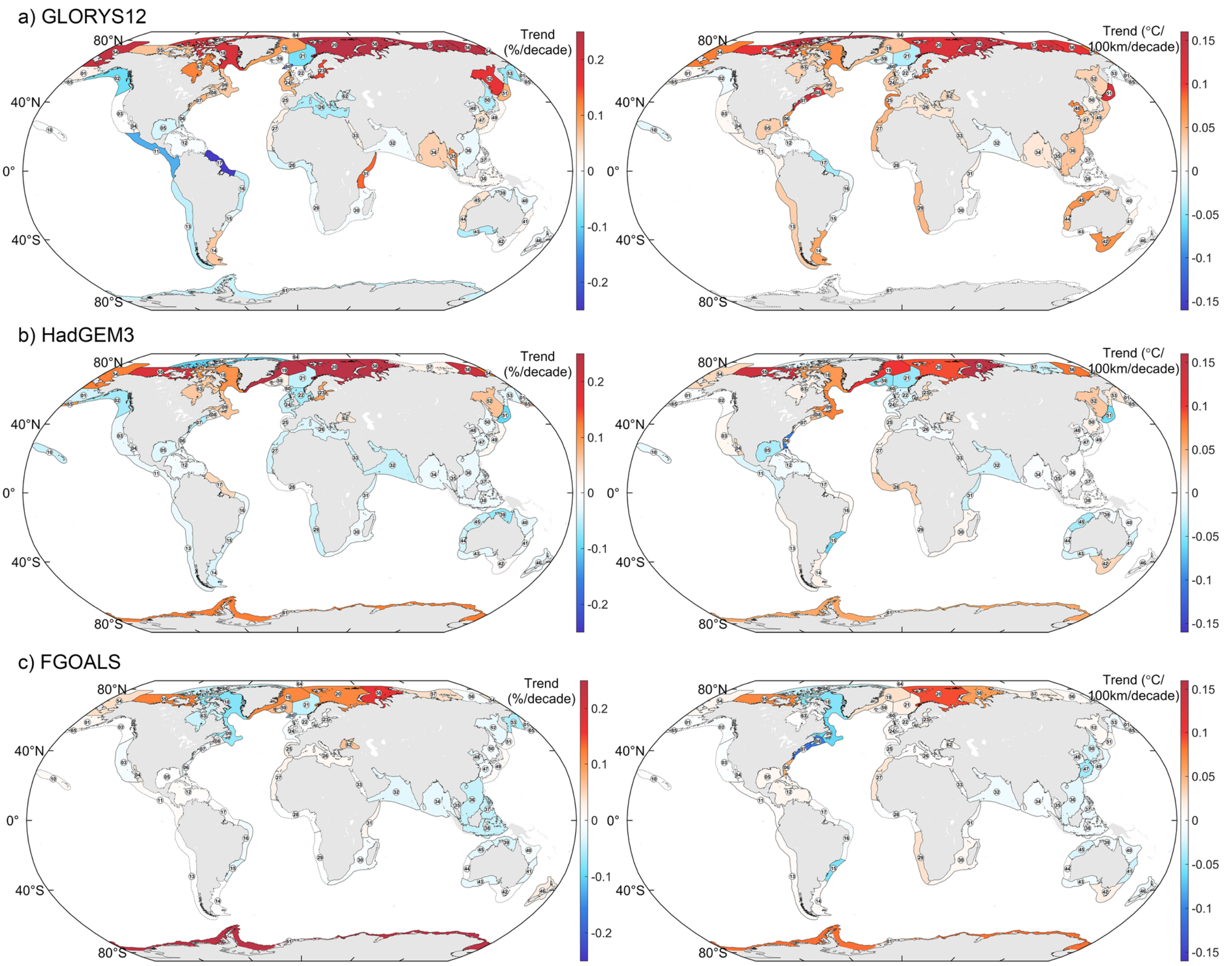

Supplementary Fig. 9 | Long-term changes in persistent fronts within Large Marine Ecosystems (LMEs) using analysis data and climate models. a, b, c, trends of persistent frontal occurrence and intensity within LMEs using analysis data GLORY12 (**a**) as well as climate models HadGEM3 (**b**) and FGOALS (**c**). Solid boundary lines of the LMEs denote statistically significant trends (*P*<0.05). The overlapping numbers within each LME represent the LME numbers.

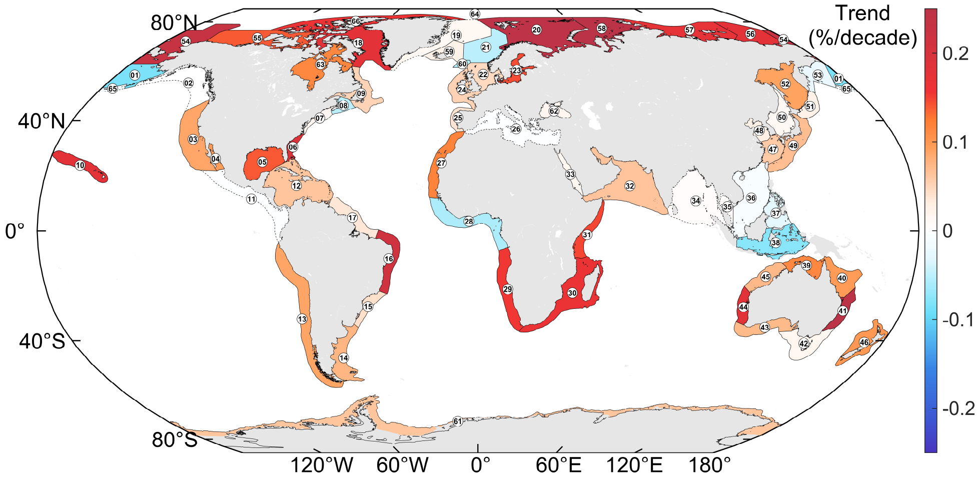

Supplementary Fig. 10 | Long-term variations of persistent fronts after disregarding sea ice-covered regions. The pixels located in daily sea ice mask were excluded when calculating monthly occurrence and intensity. Solid boundary lines of the Large Marine Ecosystems (LMEs) denote statistically significant trends ($P<0.05$). The overlapping numbers within each LME represent the LME numbers.

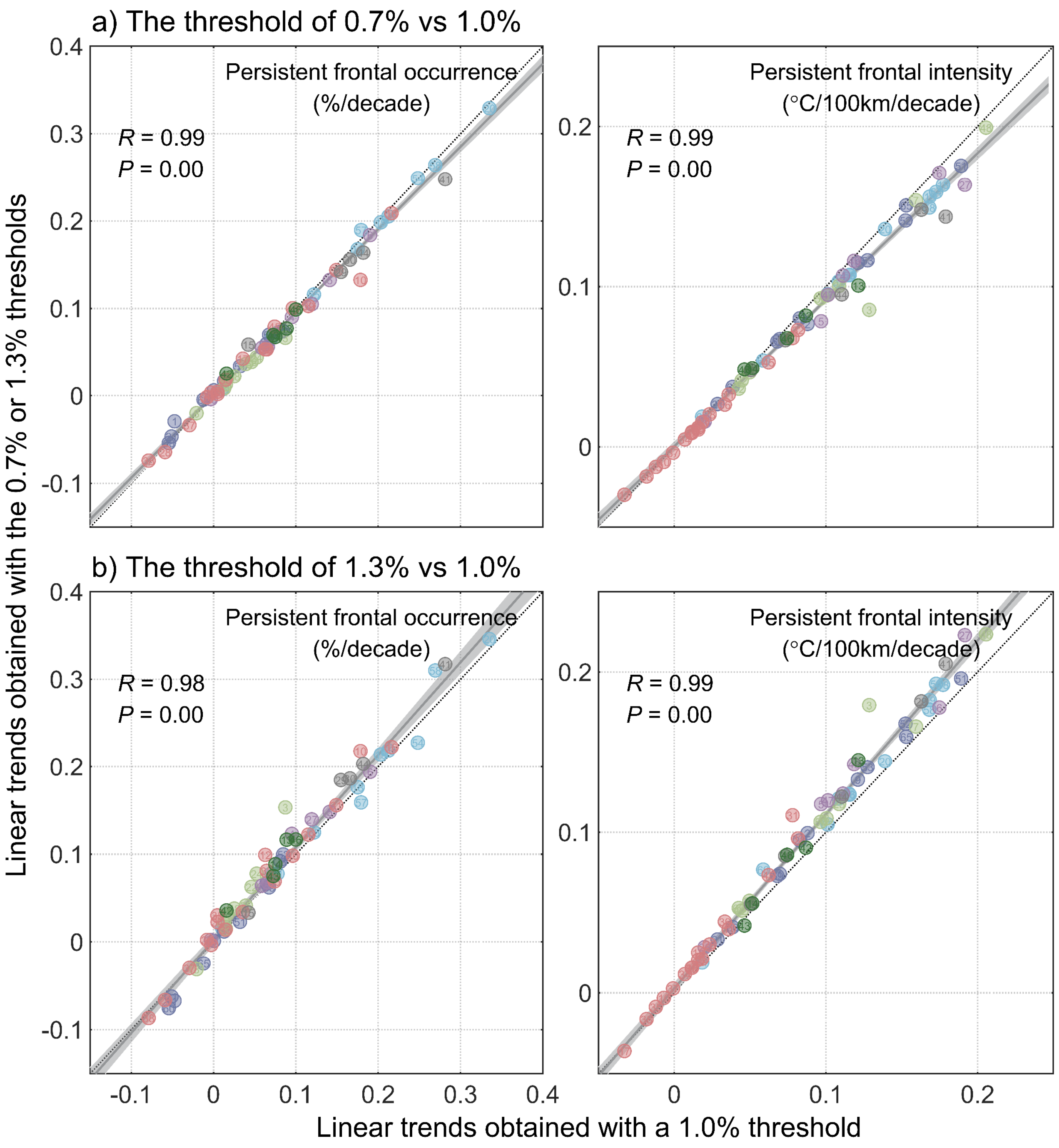

Supplementary Fig. 11 | Sensitivity analysis of threshold values for persistent frontal changes. **a**, **b**, Linear trend comparisons of the occurrence and intensity of persistent fronts between the results obtained with a 1.0% threshold and those with thresholds of 0.7% (**a**) and 1.3% (**b**) in each Large Marine Ecosystem. Thick black lines show linear regression results with 95% confidence intervals in light gray shadings, and dotted lines indicate fitted lines when the two results are nearly equal. The various colors of scatter diagrams correspond to different regions same as Fig. 1b.

Supplementary Table 1 | Detailed information on persistent fronts identified by this study. The IDs correspond to each persistent front as depicted in Fig. 1a, while single and double asterisks denote the significance of relative trend at the 0.1 and 0.01 levels, respectively.

ID	Long name	Quarter	LME number	Average occurrence (%)	Average intensity (℃/100 km)	Relative trend (%)	
						Occurrence	Intensity
1	Bohai Sea Coastal Front	1,2,3,4	48	5.13	3.59	6.06**	8.80**
2	East Bohai Sea Front	1,3,4	48	3.21	3.00	-5.29*	4.01**
3	Summer Bohai Sea Front	2	48	3.48	3.48	2.81	3.63*
4	Yantai-Weihai Coastal Front	1,2,3,4	48	3.51	2.72	-2.25*	5.36**
5	Korea Bay Coastal Front	2,3,4	48	4.52	4.13	0.35	11.19**
6	Spring Korea Bay Coastal Front	1	48	3.41	2.66	-0.34	2.44*
7	Central Yellow Sea Front	1,3,4	48	2.84	1.98	-7.95**	0.96
8	West Korean Peninsula Front	1,2,3,4	48	3.61	3.02	-1.08	7.42**
9	Gyeonggi Bay Front	1,2,3,4	48	3.40	2.83	-5.95**	11.26**
10	Winter Gyeonggi Bay Front	4	48	3.77	3.02	-14.17**	12.14**
11	Southwest Korea Front	1,2,3,4	48	3.22	2.93	4.61**	11.38**
12	Haizhou Bay Coastal Front	1,2,3,4	48	4.13	2.70	-0.43	7.39**
13	Spring Haizhou Bay Offshore Front	1	48	3.09	2.09	-4.32	-1.17
14	North Jiangsu Coastal Front	1,3,4	48	3.31	2.15	-0.44	5.04**
15	Jiangsu Shoal Front	1,2,3,4	48	3.49	2.42	-0.18	0.95
16	South Jiangsu Coastal Front	1,2,3,4	48	3.17	2.69	-7.82**	-0.35
17	South Korean Peninsula Front	1,2,3,4	47	4.73	3.24	1.27*	7.31**
18	East Jeju Coastal Front	1,2,3	47	5.05	3.64	0.87	6.25**
19	Yellow Sea Warm Current Front	1,4	48	4.87	3.44	-0.8	3.06*
20	Hangzhou Bay Front	1,2,3,4	47	3.89	2.62	-4.26**	-0.24
21	Zhejiang-Fujian Front	1,2,3,4	47	4.25	3.31	0.46	4.49**
22	Summer Zhejiang Coastal Front	2	47	2.69	1.98	-4.92	0.5
23	North Yangtze Bank Front	1,3,4	47	3.34	2.32	0.8	1.65*
24	Central Yangtze Bank Front	1,2,3,4	47	3.08	1.94	3.84*	1.82*
25	West Yangtze Bank Front	1,4	47	3.66	2.43	2.75*	3.08**
26	South Yangtze Bank Front	1,3,4	47	3.09	2.06	8.57*	2.70*
27	Central East China Sea Front	1,4	47	3.62	2.80	-3.31	-0.16
28	Kuroshio Tsushima Branch Front	1,2,4	47	3.09	2.74	4.35	3.76**
29	South Tsushima Warm Current Front	1,4	47	2.68	1.72	-5.89**	0.57
30	West Kyushu Coastal Front	1,2,3,4	47	2.48	2.00	-9.19**	6.24**
31	East China Sea Kuroshio Front	1,2,3,4	47	4.42	2.28	2.01*	3.52**
32	Kuroshio Countercurrent-Ryukyu Current Ring Front	1,2,3,4	49	2.78	0.98	6.49**	4.17**
33	Spring Ryukyu Islands Front	1	47	2.81	1.23	11.00*	7.54**
34	South Ryukyu Islands Front	1,2,3,4	49	2.19	0.78	6.15**	1.89*

35	Summer Taiwan Island Kuroshio Current Front	2	47	3.42	1.36	11.12**	4.53**
36	West Taiwan Island Front	1,2,3,4	36	3.04	2.59	2.66	3.24**
37	Central Taiwan Strait Front	1,4	36	5.31	4.76	0.22	6.60**
38	Taiwan Shoal Front	1,2,3,4	36	5.39	3.20	1.46	5.04**
39	Guangdong Coastal Front	1,2,3,4	36	4.85	2.45	2.04*	6.69**
40	Summer Guangdong Coastal Front	2	36	3.39	1.66	-0.5	4.76*
41	South China Sea Shelf Front	1,2	36	2.41	0.94	0.25	-4.60*
42	East Hainan Coastal Front	1,2,3,4	36	3.32	1.64	4.22**	4.15**
43	West Hainan Coastal Front	1,2,3,4	36	2.77	1.70	1.79	0.34
44	Beibu Gulf Coastal Front	1,2,3,4	36	3.06	1.55	-2.76*	4.02**
45	Central Beibu Gulf Front	1,4	36	4.53	2.46	2.93*	5.95**
46	East Vietnam Coastal Front	1,2,3,4	36	4.24	1.44	3.20*	5.01**
47	Xisha Islands Front	3,4	36	2.53	0.71	1.2	-2.02
48	South China Sea Jet Front	1,3,4	36	2.87	0.79	5.33*	1.22
49	South Vietnam Coastal Front	1,2,3,4	36	3.92	1.22	2.35*	3.91*
50	Peninsular Malaysia Offshore Front	1,4	35	4.07	0.95	2.16	-1.89
51	Gulf of Thailand Coastal Front	1,2,3,4	35	3.06	0.79	-1.38	-1.86
52	Bangkok Bay Front	1,2,3,4	35	3.60	0.82	5.46**	2.87*
53	Summer Gulf of Thailand Front	2	35	2.88	0.57	4.96	-2.04*
54	East Malaysia Coastal Front	1,2,3	36	3.15	0.72	0.18	-3.10*
55	Natuna Islands Front	3,4	36	2.82	0.70	2.79	0.94
56	Summer Kalimantan Strait Front	2	36	3.02	0.53	8.14*	-1.82
57	West Summer Kalimantan Strait Front	2	36	2.44	0.49	11.36**	-2.88
58	Kalimantan Island Coastal Front	1,2,3,4	36	3.15	0.88	-6.15**	-3.62**
59	Winter Kalimantan Offshore Front	4	36	3.24	0.80	-9.35**	-0.9
60	Winter North Kalimantan Offshore Front	4	36	2.60	1.00	-5.23	-5.54
61	Palawan Coastal Front	2,3,4	36	2.23	0.68	-4.15*	-3.09*
62	South Luzon Offshore Front	1,3,4	36	2.53	0.76	0.2	-3.06**
63	West Luzon Offshore Front	1,3,4	36	3.37	1.19	2.36*	0.6
64	Summer Luzon Coastal Front	2	36	2.92	0.83	0.35	-1.66
65	Kuroshio Intrusion Front	1,2,3,4	36	3.00	1.12	5.74**	1.08
66	West Batanes Front	1,2,3,4	49	2.58	1.05	6.33**	3.18*
67	East Batanes Front	2,3	49	2.09	0.81	2.79	-0.41
68	South Taiwan Island Front	1,2,3,4	49	2.00	1.06	9.74**	4.24**
69	East Taiwan Island Offshore Front	1,4	49	2.74	1.09	3.52	-0.02
70	Bangka Island Front	1,2,3,4	36	2.40	0.61	-2	-3.47*
71	West Kalimantan Island Front	1,2,3,4	36	2.96	0.77	-3.47*	-1.87
72	Belitung Island Front	1,2,3,4	38	1.97	0.55	-3.50**	-3.90*
73	South Kalimantan Island Front	1,2,3,4	38	3.20	0.77	-6.50**	-4.78**
74	West Java Sea Front	1,2,3,4	38	2.32	0.60	-2.68	-4.89**
75	South Java Sea Front	1,2,3,4	38	2.50	0.71	-6.83**	-3.75**
76	South Makassar Strait Front	1,2,3,4	38	2.85	0.83	-0.64	-1.38
77	East Java Sea Front	1,2,3,4	38	2.41	0.87	-0.86	-1.32
78	Winter East Java Sea Front	2	38	2.09	0.71	-3.32	-6.61**
79	Makassar Strait Front	1,2,3,4	38	2.22	0.73	-8.02*	-4.13*

80	Summer Flores Sea Front	4	38	1.89	0.91	-4.53	2.28
81	Winter Flores Sea Front	2	38	2.70	0.71	5.95*	-5.11*
82	Winter Alor Archipelago Offshore Front	2	38	3.07	0.74	13.39**	0.42
83	Wetar Offshore Front	1,2,3	38	2.56	0.65	1.81	-6.02**
84	Gulf of Boni Front	1,2,3,4	38	2.41	0.76	-3.87**	-3.19*
85	Selayar Island Front	1,2,3,4	38	1.87	0.84	-4.91*	-0.91
86	South Sulawesi Tenggara Front	1,2,4	38	2.24	0.77	-4.29*	-5.50**
87	Sulawesi Tenggara-Tengah Front	1,2,3,4	38	2.61	0.79	-7.70*	-4.91
88	Kepulauan Banggai-Sula Front	1,3,4	38	2.89	1.04	-4.46	0.74
89	West Molucca Sea Front	1,2,3,4	38	2.65	1.09	-4.44*	-2.26
90	Gulf of Tomini Front	2,3	38	3.06	0.95	0.3	-1.74
91	Summer Gulf of Tomini Front	4	38	1.98	0.67	-2.71	-0.12
92	West Sula Islands Front	2,3	38	2.79	1.19	9.87	3.48**
93	East Sula Islands Front	2	38	2.32	0.75	-0.53	-6.64*
94	Buru Island Front	1,2,3	38	2.65	0.88	2.96	0.47
95	Central Ceram Sea Front	2,3	38	2.37	0.90	3.95	-0.98
96	Summer Ceram Sea Front	4	38	2.57	0.75	-6.39	-0.85
97	Manipa Island Front	1,2,3,4	38	2.92	1.03	3.24*	0.71
98	East Ceram Sea Front	2,3	38	2.37	0.89	2.92	0.64
99	North Banda Sea Coastal Front	1,4	38	2.40	0.67	-1.76	-2.3
100	Birds Head-Bomberai Peninsula Front	1,2,3,4	38	3.31	1.00	-6.35*	-4.70*
101	Winter East Halmahera Sea Front	2	38	2.57	0.97	3.69	0.04
102	West Halmahera Sea Front	1,2,3,4	38	1.95	0.74	-10.97**	-5.59*
103	East Molucca Sea Front	1,2,3,4	38	2.18	0.80	-6.74**	-5.30*
104	North Molucca Sea Front	2,3	37	3.25	1.00	2.33	0.64
105	Winter North Molucca Sea Front	4	38	2.88	0.76	-1.2	-3.39*
106	North Birds Head Peninsula Front	1,3,4		2.31	0.62	-9.99**	-5.85**
107	Cenderawasih Bay Front	1,2,3,4		1.76	0.67	-2.98	-0.19
108	North New Guinea Coastal Front	1,2,3,4		3.03	0.70	-2.80*	-1.14
109	Winter Dogreto Bay Front	2		3.16	0.56	16.38**	3.17
110	Manus Island Front	2,3,4		2.13	0.51	-5.41*	-5.26**
111	North Bismarck Archipelago Front	1,4		2.25	0.59	-3.79	0.41
112	West New Ireland Front	2,3		2.45	0.79	-1.06	-0.49
113	West New Guinea Coastal Front	1,3,4	38	2.97	0.89	-6.66**	-2.29
114	Tanimbar Islands Front	1,2,3	38	2.03	0.60	2.44	-4.98**
115	Aru Islands Ring Front	1,2,3,4	38	2.09	0.86	-6.56*	-2.24
116	Winter North Arafura Sea Front	2		2.88	0.74	4.85	-1.75
117	Winter West New Guinea Front	2	38	3.09	0.87	0.69	-0.39
118	Summer East Aru Islands Front	4		2.59	0.69	2.64	0.9
119	East Arafura Sea Coastal Front	1,2,3,4	40	2.69	0.73	-2.81*	-2.90*
120	Gulf of Papua Front	1,2,3,4		3.26	0.92	-2.54*	-2.44
121	Calvados Chain Front	1,2,3,4		4.70	1.13	2.88*	4.19**
122	South Calvados Chain Front	1,4		2.84	0.85	6.31*	4.60**
123	Winter East Calvados Chain Front	2		2.87	0.66	6.35*	3.28
124	West Solomon Sea Front	1,2,3,4		2.57	0.73	-1.31	-0.81

125	North Solomon Sea Front	1,4		2.17	0.58	-2.07	-1.8
126	East Solomon Sea Front	1,2,3,4		2.74	0.63	-6.70**	-4.33*
127	North Solomon Islands Front	1,2,3		1.98	0.62	-4.99	-2.75
128	Winter Rennell Island Front	2		2.98	0.61	9.31*	2.02
129	South Rennell Island Front	2,3,4		2.42	0.53	1.03	-0.02
130	West Celebes Sea Coastal Front	1,2,3,4	37	3.12	1.05	-8.72**	-4.06*
131	Summer Derawan Islands Front	2	37	2.07	0.70	2.35	-1.98
132	South Celebes Sea Front	1,2	37	1.72	0.70	-1.93	-2.58
133	East Celebes Sea Front	1,2,3,4	37	2.55	0.71	2.07	-1.49
134	Sulu Archipelago Ring Front	1,2,3,4	37	3.55	1.21	3.53**	5.41**
135	Winter South Sulu Archipelago Front	4	37	2.83	0.81	13.89**	6.26**
136	South Mindanao Front	1,4	37	3.18	0.90	2.88	-0.79
137	South Sulu Sea Coastal Front	1,2,3,4	37	3.41	1.03	-3.43	-3.66*
138	Negros Island Offshore Front	1,4	37	2.68	1.23	-6.02*	-3.32
139	Summer North Zamboanga Peninsula Front	2	37	1.68	0.68	-0.22	-0.86
140	North Mindanao Coastal Front	1,2,3,4	37	1.83	0.87	-4.47*	-2.71
141	East Mindanao Front	1,2,3,4	37	2.27	0.61	0.69	-1.98
142	Dinagat-Samar Island Front	1,2,3,4	37	1.84	0.65	2.11	-0.57
143	West Panay Offshore Front	1,3,4	37	3.13	0.92	3.53	-1.69
144	East Calamian Islands Front	1,2,3,4	37	3.01	0.90	-1.5	-2
145	Winter East Palawan Island Front	4	37	1.99	0.73	-11.19**	-6.88**
146	Winter Panay Bay Front	4	37	1.73	0.75	8.49	-4.84**
147	Visayan Sea Front	1,2,3,4	37	1.47	0.96	-11.80**	-1.49
148	Sibuyan Sea Front	3,4	37	1.86	0.80	4.51*	-0.94
149	North Samar Front	1,2,3,4		1.48	0.92	-2.73	3.88**
150	North Bicol Peninsula Front	1,2,3,4		1.84	0.67	-10.87**	-3.33*
151	Winter Polillo Islands Offshore Front	4		2.58	0.62	14.24*	2.32
152	East Luzon Front	1,2,3,4		2.40	0.71	-1.88	-1.31
153	West Sumatra Island Front	1,2,3,4	34	2.44	0.66	-0.89	-0.03
154	South Java Island Front	1,2,3,4		3.09	0.97	1.42	1.97
155	Bali Island Offshore Front	2,3	38	3.29	1.25	6.53*	5.31*
156	Summer South Nusa Tenggara Front	4		2.49	0.86	-14.65**	0.3
157	Savu Sea Front	1,2,3	38	1.89	1.10	1.64	1.35
158	Summer South Sumba Front	4	38	2.39	0.73	-4.16	-1.52
159	Timor Island Front	1,2,3,4		2.50	0.70	5.59**	-1.42
160	Summer East Timor Island Front	4	38	2.88	0.72	1.15	0.6
161	Gulf of Carpentaria Front	1,2,3,4	39	3.87	1.17	3.31**	2.04**
162	Spring Gulf of Carpentaria Front	3	39	2.74	0.76	14.34**	-1.1
163	Groote Eylandt Front	1,2,3,4	39	2.45	0.94	2.8	-0.83
164	South Arafura Sea Front	1,2,3,4	39	3.90	0.97	3.23*	-0.58
165	West Tiwi Islands Offshore Front	1,3,4	39	2.23	0.70	5.45*	-1.67
166	East Timor Sea Front	2,3	39	2.72	0.60	9.61*	-2.55
167	Winter East Timor Sea Front	2	39	2.19	0.46	-2.27	-6.96*
168	Van Diemen Gulf Front	1,2,3	39	2.90	0.96	5.16*	-1.32
169	Joseph Bonaparte Gulf Front	1,2,3,4	39	3.93	1.34	-0.15	0.74

170	South Joseph Bonaparte Gulf Front	1,2,3	39	2.82	1.51	-4.1	2.13*
171	Kimberley Coastal Front	1,2,3,4	45	3.53	1.26	-2.24*	0.78
172	North Kimberley Offshore Front	1,2,3,4	45	2.65	0.84	2.86	-0.09
173	Autumn Ashmore Reef Front	1	45	2.31	0.52	7.33	-3.2
174	Buccaneer Archipelago Front	1,2,3,4	45	2.55	0.97	2.33	-0.65
175	West Kimberley Offshore Front	1,3,4	45	2.67	0.72	5.79*	-1.54
176	Winter Rowley Shoals Front	2	45	2.88	0.61	4.85	0.27
177	King Sound Front	1,2,3,4	45	3.05	1.49	5.48**	2.20*
178	West Pilbara Coastal Front	1,2,3,4	45	5.95	2.42	1.37**	5.53**
179	West Pilbara Offshore Front	1,2,3	45	2.39	0.87	3.21	1.93*
180	Ningaloo Coast Front	1,2,3,4	44	2.39	1.43	5.60**	5.59**
181	Shark Bay Front	1,2,3,4	44	5.82	3.18	0.75	7.57**
182	West Australian Coastal Current Front	1,2,3,4	44	4.01	1.72	5.05**	7.37**
183	Leeuwin Current Front	1,2,3,4	44	3.60	1.48	4.64**	4.02**
184	Leeuwin Current Extension Front	1,2,3,4	43	4.97	1.53	-0.65	2.27*
185	Diamantina Fracture Zone Front	1,2,3,4	43	3.73	1.49	0.12	5.63**
186	Great Australian Bight Coastal Front	1,2,3,4	43	4.69	1.20	3.99**	5.87**
187	Autumn Great Australian Bight Front	1	43	4.70	0.99	-3.53	-2.59
188	Cednua Sub-Basin Front	1,2,4	43	3.66	0.98	7.77**	4.12*
189	Spencer Gulf Front	1,2,3,4	43	3.88	1.65	2.93**	3.15**
190	East Spencer Gulf Front	1,2,3,4	43	2.44	1.37	4.23**	4.82**
191	Gulf St Vincent Front	1,2,3,4	43	2.78	1.62	5.16**	4.42**
192	Encounter Bay Front	1,2,3	42	3.54	1.57	1.38*	4.57**
193	Summer Encounter Bay Front	4	42	4.63	2.16	-9.69**	2.24
194	West Victoria State Coastal Front	1,2,3,4	42	4.25	1.22	4.25*	6.06**
195	East Great Australian Bight Offshore Front	1,2,3,4	42	3.54	0.98	-0.64	4.66*
196	Summer West Tasmania Offshore Front	4	42	3.47	0.98	1.7	2.99
197	King Island Front	1,2,3,4	42	2.08	0.92	-6.96**	-1.98
198	South Bass Strait Coastal Front	1,2,3,4	42	3.03	1.05	3.48*	4.35**
199	Spring Bass Strait Front	3	42	2.27	0.95	5.68	4.55*
200	North Bass Strait Coastal Front	1,2,3,4	42	3.79	1.69	0.51	9.49**
201	South Tasman Rise Front	1,2,3,4	42	3.80	1.40	0.57	9.85**
202	Tasman Basin Front	1,2	42	3.58	1.58	-0.56	6.29*
203	Tasman Plateau Front	1,4	42	3.71	1.79	2.67	6.53**
204	East Australia Current Extension Front	1,2,3,4	42	5.03	2.19	4.59**	8.64**
205	Autumn East Furneaux Group Offshore Front	1	42	3.39	1.56	0.05	-0.22
206	East Australia Current Front	1,2,3,4	41	5.63	2.33	4.25**	7.15**
207	Fraser Island Front	1,2	41	4.99	2.48	3.71*	10.24**
208	South Great Barrier Reef Front	1,2,3,4	40	4.39	1.02	4.41**	0.06
209	East Queensland Coastal Front	1,2,3,4	40	4.26	1.43	1.12	3.30**
210	Lake Shoal Coastal Front	3,4	40	3.36	0.90	-1.46	-5.31**
211	Capricorn and Bunker Group Front	3,4	40	2.89	0.96	2.61	-1.68
212	North Great Barrier Reef Front	1,2,3,4	40	3.71	1.00	0.45	1.19
213	Princess Charlotte Bay Front	1,2,3,4	40	5.00	1.25	3.59*	2.49*
214	Winter Cape York Front	2	40	2.83	0.70	2.11	-1.8

215	Winter East Cape York Front	2	40	2.61	0.63	8.21	3.50*
216	East Cape York Peninsula Front	1,2,4	40	2.21	0.59	9.84**	0.87
217	Tregosse and Lihou Reefs Front	1,2,3,4	40	2.89	0.64	2.4	-0.65
218	West New Caledonia Front	1,2,3,4		3.22	0.82	4.92**	0.7
219	Chesterfield-Bellona Plateau Front	1,2,3		3.05	0.78	4.28	1.28
220	New Caledonia Front	1,2,3,4		2.81	1.01	1.25	1.56
221	Vanuatu Islands Front	1,2,3,4		2.94	0.79	1.96	-2.87*
222	Wintter South Vanuatu Islands Front	2		3.31	0.91	5.81	1.17
223	Fiji Front	1,2,3,4		2.86	0.86	4.17**	-0.45
224	West Fiji Front	1,2,3,4		2.75	0.86	-1.7	-5.24**
225	South Fiji Front	1,2,3,4		3.08	0.87	-0.35	-2.79
226	Tasman Front	1,2,3,4	46	3.94	1.29	0.13	0.59
227	East Auckland Current Front	1,2,3,4	46	4.94	1.74	0.7	4.29**
228	Hauraki Gulf Front	1,2	46	2.65	1.67	-6.36*	6.36**
229	East Cape Current Front	1,2,3,4	46	4.86	1.67	0.17	3.53*
230	Hawke Bay Front	1,2,3,4	46	4.13	1.52	4.05	4.24**
231	Southland Current Front	1,2,3,4	46	6.04	2.04	1.91**	6.58**
232	Southland Current Extension Front	1,2,3,4		3.89	1.01	0.79	2.71*
233	East Cape Current Branch Front	1,2,3,4		3.72	1.06	1.49	-0.27
234	Winter Canterbury Bight Coastal Front	2	46	6.15	1.74	4.76*	10.21**
235	Pegasus Bay Front	1,2,4	46	3.66	1.78	-4.91*	0.14
236	West Chatham Rise Front	1,2,3,4	46	4.47	1.92	0.4	1.92
237	Cook Strait Front	1,2,3,4	46	3.65	2.10	0.77	5.42**
238	South Taranaki Bight Front	1,2,3,4	46	3.88	1.61	4.11**	5.23**
239	West Auckland Current Front	1,2,3,4	46	3.14	1.23	8.73**	6.93**
240	Challenger Plateau Front	1,2,3,4	46	4.55	0.98	2.1	2.62
241	West Challenger Plateau Front	1,2	46	3.56	0.76	3.84	2.19
242	East Tasman Sea Front	1,2,3		3.71	1.01	-0.45	4.79**
243	Westland Current Front	1,2,3,4	46	3.95	1.56	4.55**	6.32**
244	Foveaux Strait Front	1,2,3,4	46	3.12	1.61	10.63**	7.79**
245	West Stewart Island Front	1,2,3,4	46	3.47	1.11	-0.14	3.14**
246	South Stewart Island Front	1,2,3,4	46	3.93	1.45	-1.4	2.56**
247	Spring Southeast Stewart Island Front	3	46	2.27	0.91	-14.01*	0.19
248	Snares Islands Front	3,4		4.23	1.19	-2.74	5.56**
249	West Auckland Islands Front	2,3,4		3.97	0.95	1.4	5.95**
250	East Auckland Islands Front	1,2,3,4		3.03	0.64	4.56*	5.70**
251	South Auckland Islands Front	1,2,3,4		2.50	0.53	-0.73	5.79**
252	McDougall Trough Ring Front	1,2,3,4		3.63	1.01	-0.19	5.38**
253	Centtral Campbell Plateau Front	1,2		4.07	0.62	-0.19	3.04*
254	Summer Centtral Campbell Plateau Front	4		3.94	0.71	1.23	0.37
255	North Campbell Plateau Front	1,2,4		2.13	0.56	14.95**	5.62**
256	Campbell Islands Front	1,2,3,4		3.43	0.56	2.43	2.77*
257	Bounty Trough Front	1,2,3,4		4.41	1.23	0.2	6.88**
258	West Bounty Trough Front	2,3,4		3.51	0.89	2.58	4.03*
259	East Bounty Islands Front	1,2,3,4		4.45	1.73	-0.79	8.98**

260	Summer South Chatham Slope Front	4		3.15	0.86	-0.43	1.64
261	South Japan Kuroshio Front	1,2,3,4	49	4.61	2.77	1.64	3.57*
262	Kuroshio Extension Front	1,2,3,4	49	4.12	3.09	-0.92	4.18*
263	South Honshu Coastal Front	1,2,3,4	49	3.16	2.57	2.97	5.60*
264	South Japan Kuroshio Outer Front	1	49	3.92	1.84	0.26	5.49**
265	West Seto Inland Sea Front	1,2,3,4	49	3.60	3.84	-1.33	6.87**
266	East Seto Inland Sea Front	1,2,3,4	49	3.96	3.64	3.62*	-0.09
267	Kashima Nada-Sendai Bay Front	1,2,3,4	49	3.03	3.04	4.22*	11.45**
268	Kashima Nada-Sendai Wan Offshore Front	1,2	49	3.64	3.78	-1.95	10.72**
269	Iwate Prefecture Offshore Front	1,2,4	49	3.55	3.58	2.3	8.13**
270	South Tsugaru Strait Front	1,4	49	3.74	4.16	2.85	5.06*
271	Autumn South Hokkaido Front	3	49	4.75	3.11	2.09	1.98
272	Cape Erimo Front	1,2,3,4	51	3.59	3.49	0.59	6.26**
273	Oyashio Current Front	1,2,3,4	51	3.78	2.29	-0.37	8.43**
274	West Oyashio Extension Front	1,2,3,4	49	4.45	2.94	-2.55*	6.94**
275	East Oyashio Extension Front	1,2,3,4		4.42	2.80	-4.25**	5.66**
276	Tsushima Warm Current Front	1,2,3,4	50	3.44	1.54	-1.81*	0.63
277	Summer South Oki Islands Front	2	50	3.72	1.56	-4.95	-2.65
278	Central Japan Sea Front	1,2,3,4	50	4.53	3.15	-2.78**	1.17
279	East Korea Bay Front	1,2,3,4	50	2.78	2.75	5.67	4.22*
280	East Korea Offshore Front	1,3,4	50	3.65	2.78	1.84	6.14*
281	West Japan Offshore Front	1,3,4	50	3.31	1.73	-5.28*	-1.59
282	West Honshu Coastal Front	1,2,3,4	50	2.44	1.91	5.62**	-0.42
283	Yamato Bank Front	1,2,3,4	50	4.72	3.22	-0.09	2.61
284	South Liman Current Front	1,2,3,4	50	3.54	2.69	0.43	7.34**
285	North Liman Current Front	1,2,3,4	50	4.25	2.32	1.13	8.23**
286	Liman Current Branch Front	1,2,3,4	50	3.57	2.58	1.43	4.39*
287	North Sea of Japan Front	1,2,3,4	50	3.90	2.48	4.49*	7.00**
288	West Hokkaido Front	1,2,4	50	2.24	2.23	4.05*	1.31
289	Autumn West Tsugaru Strait Front	3	50	4.23	3.03	-5.57	-8.80**
290	Summer West Soya Strait Front	2	50	3.31	2.76	-8.42*	2.35
291	West Karafuto Front	1,2,3,4	50	2.84	1.95	2.01	5.40**
292	Spring West Sakhalin Front	1	50	4.30	2.33	7.57*	6.10*
293	Autumn West Sakhalin Front	3	50	3.11	2.39	-2.92	3.18
294	West Strait of Tartary Front	1,2	50	1.85	1.54	6.59*	4.93*
295	Soya Warm Current Front	1,2,3,4	52	3.93	3.01	0.57	4.63**
296	South Kuril Islands Front	1,2,3,4	51	3.54	2.39	7.53**	12.17**
297	North Kuril Islands Front	1,2,3,4	52	2.90	1.59	4.65*	15.87**
298	Spring Central Kuril Islands Front	1	51	3.81	1.16	4.69	8.85*
299	Autumn West Iturup Front	3	52	3.24	2.83	0.28	5.48**
300	Spring Aniva Bay Front	1	52	2.39	1.36	-7.35*	2.9
301	Autumn East Aniva Bay Front	3	52	2.63	1.81	1.87	5.79*
302	Gulf of Patience Front	1,2,3,4	52	2.54	1.95	1.09	7.25**
303	Winter North Kuril Basin Front	4	52	3.39	0.77	-0.55	3.35
304	East Gulf of Patience Front	1,2,3,4	52	2.60	1.44	3.97*	7.91**

305	Winter West Kuril Islands Front	4	52	3.75	1.27	-0.09	7.43**
306	East Sakhalin Current Offshore Front	1,2,3,4	52	2.20	1.22	4.01*	9.12**
307	East Sakhalin Coastal Front	2,3,4	52	1.89	1.69	-0.87	11.88**
308	Sakhalin Gulf Front	1,2,3,4	52	2.19	2.49	1.44	8.43**
309	Sakhalin Gulf Offshore Front	2,3	52	3.69	3.40	-1.07	5.84**
310	Shantar Islands Front	2,3	52	4.16	3.23	0.35	6.94**
311	Academy Bay Front	2,3	52	2.60	4.25	0.43	6.35**
312	Kashevarov Bank Ring Front	1,2,3,4	52	3.72	1.89	7.02**	18.05**
313	West Kashevarov Bank Front	2,3,4	52	2.57	1.48	-1.8	10.04**
314	North Okhotsk Rise Front	1,3,4	52	3.74	0.81	-0.92	5.99**
315	Winter East Deryugin Basin Front	1,4	52	1.55	0.38	2.42	8.44**
316	Institut Okeanologii Rise Front	1,4	52	2.78	0.63	9.27**	12.48**
317	North Okhotsk Current Coastal Front	1,2,3,4	52	3.16	1.37	4.59**	9.05**
318	North Okhotsk Current Offshore Front	2,3,4	52	3.25	1.64	4.09**	9.05**
319	West Kamchatka Current Front	1,2,3,4	52	4.12	1.60	-0.09	8.85**
320	South Kamchatka Peninsula Offshore Front	2,3	52	2.47	1.48	-4.55*	8.30**
321	East Shelikhov Gulf Front	1,2,3,4	52	3.14	1.66	0.61	7.66**
322	South Shelikhov Gulf Front	1,2,3,4	52	3.49	1.69	1.37	10.73**
323	West Shelikhov Gulf Offshore Front	2,3,4	52	3.20	1.98	2.20*	7.59**
324	Gizhigin Bay Front	2,3	52	4.60	2.37	2.78	6.35**
325	Yam Bay Front	2,3	52	2.73	2.54	-2.47	6.59**
326	North Penzhina Bay Front	1,2,3	52	3.41	2.37	9.71**	9.93**
327	South Kamchatka Current Coastal Front	1,2,3,4	53	3.28	1.63	1.15	9.88**
328	South Kamchatka Current Offshore Front	1,3,4	53	3.72	1.33	-1.32	4.66*
329	North Kamchatka Current Front	1,2,3,4	53	5.04	2.08	0.67	8.28**
330	Kamchatskiy Zaliv Front	1,2,3,4	53	2.78	1.67	5.81*	9.28**
331	Karaginsky Gulf Front	3,4	53	2.84	1.43	8.41**	10.26**
332	Summer South Karaginsky Gulf Front	2	53	3.63	3.12	0.29	4.73**
333	North Karaginsky Gulf Front	2,3	53	3.19	2.55	-5.44*	1.62
334	Spring Olyutor Gulf Offshore Front	1	53	3.19	0.96	-2.94	2.62
335	Winter North Aleutian Basin Slope Front	4	53	2.34	0.43	12.71*	11.06**
336	West Aleutian Islands Front	1,2,3,4	53	3.43	0.92	-4.76**	5.35**
337	Komandor Basin Front	3,4	53	2.31	0.68	1.5	8.36**
338	East Shirshov Ridge Front	1,3,4	53	3.33	0.61	-2.25	4.23*
339	Central Aleutian Islands Front	1,2,3,4	65	3.52	0.78	0.49	13.60**
340	Bowers Ridge Front	1,3,4	1	3.76	0.58	-2.27	5.94**
341	South Bowers Ridge Front	1,4	1	2.58	0.41	-4.65	4.07*
342	Aleutian Northern Slope Curren Front	1,2,3,4	65	3.37	0.89	3.59*	16.48**
343	East Aleutian Islands Front	1,2,3,4	65	4.54	1.22	-0.24	14.50**
344	Central Aleutian Basin Front	1,3,4	1	2.26	0.56	0.51	3.42*
345	Bering Slope Current Front	1,2,3,4	1	3.94	1.10	-4.18**	2.33*
346	East Bering Sea Outer Shelf Front	1,3,4	1	3.80	1.13	-5.73**	2.12
347	North Alaska Peninsula Offshore Front	1,2,3,4	1	4.85	1.66	-5.93**	1.04
348	East Bering Sea Mid-Shelf Front	1,2,3,4	1	3.75	1.30	-0.97	5.48**
349	East Bering Sea Inner Shelf Front	2,3,4	1	3.46	1.72	-4.10*	4.01*

350	North Alaska Peninsula Coastal Front	1,2,3,4	1	2.62	1.75	12.60**	6.29**
351	North Bristol Bay Coastal Front	1,2,3,4	1	4.34	2.70	1.92	6.43**
352	North Nunivak Island Front	2,3	54	3.74	2.69	0.31	6.89**
353	South St Matthew Island Front	1,2,3,4	1	3.41	1.20	-5.88	4.99*
354	North St Matthew Island Front	2,3,4	1	2.34	0.86	-1.28	7.90**
355	South St Lawrence Island Front	2,3,4	54	2.44	1.30	3.69	11.94**
356	Norton Sound Front	1,2,3	54	2.75	1.83	3.65**	7.56**
357	South Gulf of Anadyr Coastal Front	2,3	54	3.37	2.26	3.58*	6.00**
358	Gulf of Anadyr Offshore Front	2,3	54	3.76	1.94	-1.08	4.79*
359	Anadyr Estuary Front	2,3	54	2.64	2.72	7.94**	11.56**
360	Kresta Bay Front	2,3	54	4.12	3.12	4.12	6.95**
361	South Chukchi Peninsula Front	2,3	54	4.79	2.62	1.29	8.85**
362	East Chukchi Peninsula Front	2,3	54	3.49	2.56	-0.61	8.38**
363	Autumn Bering Strait Front	3	54	3.57	1.94	-2.24	4.24
364	North Bering Strait Front	2,3	54	3.37	2.12	-2.9	2.66
365	North Chukchi Peninsula Front	2,3	54	3.58	1.78	7.85**	7.92**
366	North Seward Peninsula Front	2,3	54	2.55	2.10	3.3	6.92**
367	North Kotzebue Sound Front	2,3	54	3.67	2.32	4.65**	9.01**
368	Herald Shoal Front	2,3	54	4.30	1.84	1.15	3.8
369	North Alaskan Coastal Current Front	2,3	54	3.67	2.10	8.99**	10.03**
370	Hanna Shoal-Alaskan Coastal Current Front	2,3	54	4.34	2.31	6.55**	7.61**
371	Anadyr Current South Branch Front	2,3	54	3.94	1.89	8.15*	9.64**
372	Bering Shelf Water Front	2,3	54	3.50	1.46	19.15**	21.77**
373	Anadyr Current North Branch Front	3	54	3.29	0.99	19.23*	25.11**
374	Wrangel Island-Herald Canyon Front	2,3	54	3.26	1.60	14.16**	17.55**
375	Summer Wrangel Island Front	2	54	2.74	0.99	17.98**	33.51**
376	Siberian Coastal Current Front	2,3	54	2.85	1.26	15.18**	21.13**
377	Autumn Chukchi Slope Current Front	3	55	3.31	1.16	11.00*	14.64*
378	West Beaufort Sea Coastal Front	2,3	55	3.00	1.70	10.14**	14.09**
379	East Beaufort Sea Coastal Front	2,3	55	3.11	2.46	0.76	6.19**
380	East Beaufort Sea Offshore Front	2,3	55	3.60	2.44	7.80**	12.03**
381	West Banks Island Offshore Front	2,3	55	2.73	1.36	8.54	11.86
382	West Banks Island Coastal Front	2,3	55	2.78	1.39	9.66*	13.64**
383	Summer West Prince Patrick Island Front	2	55	2.08	0.63	15.95*	21.56*
384	North Amundsen Gulf Front	2,3	55	2.93	1.58	8.41*	10.70*
385	South Amundsen Gulf Front	2,3	55	2.87	1.47	0.92	5.03*
386	Prince Albert Sound Front	2,3	55	2.10	1.10	5.92*	10.80**
387	Coronation Gulf Front	2,3	55	2.64	1.58	14.33**	14.28**
388	Autumn Coronation Gulf Front	3	55	3.27	0.93	3.46	9.29**
389	South Queen Maud Gulf Front	2,3	55	2.04	1.27	13.77**	15.15**
390	North Queen Maud Gulf Front	2,3	55	2.87	1.55	9.18**	12.87**
391	Alaskan Stream Front	1,2,3,4	2	3.69	0.91	-0.85	7.61**
392	Alaskan Current Front	1,3,4	2	3.33	0.81	-2.5	2.06
393	South Alaska Peninsula Offshore Front	1,2,3,4	2	3.71	1.00	-1.82	4.39**
394	East Kodiak Island Front	1,2,3,4	2	2.91	1.09	-3.58**	4.36**

395	South Alaska Peninsula Coastal Front	1,2,3,4	2	3.14	1.81	2.16**	8.23**
396	Unimak Island Front	1,4	2	3.04	1.57	3.75	3.17
397	Summer Shumagin Islands Front	2	2	3.03	1.69	-2.91	7.86**
398	Chignik Coastal Front	2,3	2	2.08	1.07	6.42	5.88**
399	West Kodiak Island Front	1,2,3,4	2	2.16	1.10	-0.08	7.48**
400	South Kenai Peninsula Offshore Front	2,3,4	2	3.35	1.37	0.5	5.72**
401	South Kenai Peninsula Coastal Front	1,2,3,4	2	3.90	1.42	-0.11	6.47**
402	Prince William Sound Front	1,2,3,4	2	3.07	1.41	-1.31	9.83**
403	Prince William Sound Offshore Front	1,2,3,4	2	4.54	1.44	2.07	10.24**
404	Yakutat Bay Offshore Front	1,2,3,4	2	4.91	1.51	4.70**	12.23**
405	Summer Yakutat Offshore Front	2	2	3.34	1.25	-5.7	2.53
406	Cross Sound Offshore Front	1,2,3,4	2	2.78	1.26	-2.32	10.07**
407	Summer South Gulf of Alaska Front	2	2	3.34	0.75	-3.53	2.34
408	Winter South Gulf of Alaska Front	4	2	2.52	0.50	11.51*	10.30**
409	Summer Kodiak-Bowie Seamount Chain Front	2	2	3.13	0.79	1.65	5.62
410	Alexander Archipelago Coastal Front	1,2,3,4	2	3.29	1.53	3.55*	8.02**
411	Alexander Archipelago Offshore Front	1,2,3,4	2	2.00	0.68	4.18	4.17*
412	North Haida Gwaii Front	1,2,3,4	2	3.28	1.21	-1.99	4.17*
413	Hecate Strait Front	1,2,3,4	2	3.00	1.62	0.38	6.53**
414	Dixon Entrance Front	1,2,3,4	2	2.84	1.42	-1.71	6.58**
415	Hecate Strait-Queen Charlotte Sound Front	1,2,3,4	2	3.85	1.59	2.47	9.29**
416	Cape Caution-Sutil Front	1,2	2	3.00	1.77	1.99	9.55**
417	Summer South Haida Gwaii Front	2	2	2.30	1.41	-0.29	7.75**
418	South Haida Gwaii Front	3,4	2	3.03	1.04	0.62	6.57**
419	Vancouver Island Offshore Front	2,3	2	3.69	2.07	-0.98	6.11**
420	Vancouver Island Coastal Front	1,4	2	3.86	1.34	-1	4.11*
421	Autumn West Washington Offshore Front	3	2	3.29	1.16	2.97	3.46
422	Winter Juan de Fuca Ridge Front	4	2	3.14	0.62	-0.03	4.42
423	North California Upwelling Front	1,2,3,4	3	3.72	2.06	1.34	8.38**
424	North California Offshore Front	2,3	3	4.02	2.75	2.64	8.48**
425	South California Offshore Front	2,3	3	3.72	2.24	3.99	7.01**
426	South California Upwelling Front	1,2,3,4	3	3.76	1.95	4.24**	7.53**
427	Winter West San Francisco Offshore Front	4	3	3.48	1.22	2.36	6.06*
428	East Channel Islands of California Front	1,2,3,4	3	3.12	1.71	2.75*	5.55**
429	South Channel Islands of California Front	1,2,3	3	3.30	1.86	-6.19**	2.44
430	Baja California Front	1,2,3,4	3	4.57	2.07	9.86**	11.68**
431	Summer Guadalupe Island Front	2,3	3	3.08	1.14	-2.78	1.55
432	Sebastián Vizcaíno Bay Front	1,3,4	3	1.67	2.10	-6.1	2.94
433	Punta Eugenia Offshore Front	1,4	3	3.09	1.36	-0.71	4.59**
434	North Baja California Sur Front	1,2,4	3	3.17	2.23	4.76*	10.48**
435	Punta Hughes Offshore Front	3,4	3	3.62	1.74	8.36**	5.34**
436	Spring Punta Hughes Offshore Front	1	3	3.28	2.20	-4.76	7.69*
437	South Baja California Sur Front	1,2,3,4	3	3.47	1.92	4.54**	4.31**
438	North Gulf of California Ring Front	1,2,3,4	4	3.17	2.26	5.24**	6.26**
439	South Gulf of California Front	1,2,3,4	4	3.59	1.76	1.65	4.71**

440	Wset Tiburón Island Front	1,2,3,4	4	3.61	2.99	1.63	3.98**
441	Isla Carmen Front	2,3,4	4	2.05	1.63	-0.4	0.13
442	West Mexico Coastal Front	1,2,3,4	11	3.62	1.36	4.07**	4.67*
443	West Guatemala-Nicaragua Coastal Front	1,2,3,4	11	3.36	1.27	1.32	0.69
444	Gulf of Tehuantepec Offshore Front	1,3,4	11	3.90	2.23	2.51	3.86
445	West Costa Rica Front	1,2,4	11	3.57	1.91	-2	4.44*
446	Winter West Nicaragua Offshore Front	4	11	3.68	1.93	3.49	4.3
447	South Panama Front	1,2,3,4	11	2.99	1.09	-1.61	2.25
448	West Gulf of Panama Front	1,4	11	3.00	1.71	3.03*	4.24*
449	East Gulf of Panama Front	1,4	11	3.07	1.58	-0.78	6.95**
450	Colombia-Ecuador Front	1,2,3,4	11	3.38	1.29	-5.98**	-0.36
451	Cocos Islands Front	2,3		2.00	0.43	0.7	0.52
452	North Humboldt Current Front	2,3,4	11	3.97	2.18	1.3	4.22*
453	East Pacific Equatorial Convergence Front	2,3,4		3.85	1.95	0.56	4.37*
454	South Equatorial Current Front	1,2,3		3.15	1.06	-4.29*	-1.8
455	South Galápagos Islands Front	2,3,4		2.95	1.69	8.61*	8.21*
456	Spring Galápagos Islands Front	1		3.01	2.15	9.67**	7.22**
457	Sechura Bay Offshore Front	1,2,3,4	13	3.72	2.45	-6.26**	3.38**
458	Middle Humboldt Current Front	1,2,3,4	13	4.74	2.53	0.48	1.92
459	Independencia Bay Front	1,2,3,4	13	3.27	2.38	-6.98**	3.97**
460	South Humboldt Current Front	1,2,3,4	13	5.46	2.83	3.74**	10.34**
461	Chile Coastal Front	1,2,3,4	13	4.21	1.76	4.32**	7.97**
462	Gulf of Ancud Front	1,2,3,4	13	3.21	1.72	1.9	5.17**
463	Gulf of Corcovado Front	1,2,3,4	13	2.77	1.24	1.61	5.93**
464	Chonos Archipelago Front	1,2,3,4	13	2.61	0.96	1.11	4.15*
465	Taitao Peninsula Front	3,4	13	2.77	0.98	-1.36	1
466	Autumn Campana Island Front	1	13	3.61	0.87	5.23	7.70**
467	South Chile Coastal Front	1,2,3	13	3.00	1.28	5.50*	7.29**
468	Cape Horn Current Front	1,2,3,4	13	6.06	1.93	1.08*	5.46**
469	West Tierra del Fuego Front	1,4	13	3.78	0.81	-1.99	1.78
470	West Cape Horn Current Front	1,2,3	13	3.52	0.78	-5.67**	4.18*
471	Cape Horn Current Extension Front	1,2,3,4		4.09	1.39	-3.06*	7.84**
472	East Malvinas Current Front	1,2,3,4	14	4.09	1.02	0.87	2.82**
473	North Burdwood Bank Front	2,3,4	14	3.41	0.86	2.46	2.39*
474	South Burdwood Bank Front	1,2,3,4	14	2.29	0.71	1.09	4.02**
475	East Malvinas Islands Front	2,3		2.83	0.68	3.42	10.50**
476	Malvinas Islands Ring Front	1,2,3,4	14	3.46	1.10	4.87**	7.84**
477	West Malvinas Current Front	1,2,3,4	14	4.23	2.13	0.62	4.00**
478	Malvinas Current Return Front	1,2,3,4		4.39	2.80	0.3	5.58**
479	Malvinas Current Return Extension Front	1,2,3,4		5.28	3.55	-3.78**	6.62**
480	South Brazil Current Front	1,2,3,4	15	4.39	2.97	2.43*	3.37*
481	Brazil Current Return Front	1,2,3,4		3.73	3.54	1.51*	5.72**
482	East Tierra del Fuego Front	1,2,3,4	14	4.50	1.55	8.55**	10.03**
483	Isla de los Estados Front	1,4	14	2.70	0.87	7.70**	0.46
484	Tierra del Fuego Offshore Front	1,3	14	2.74	0.77	-4.46	0.89

485	Bahía Grande-Malvinas Front	1,2,3,4	14	2.87	0.82	0.43	0.94
486	South Argentina Offshore Front	1,2	14	2.75	0.89	-1.03	2.21
487	West Malvinas Current Branch Front	3,4	14	2.66	1.30	-1.4	-0.68
488	San Jorge Gulf-Bahía Grande Front	1,2,3,4	14	3.95	1.38	1.82**	4.97**
489	Summer South Argentina Offshore Front	4	14	3.49	1.33	0.69	1.8
490	Spring San Jorge Gulf Front	3	14	5.06	1.40	-4.35*	-3.62*
491	San Jorge Gulf Front	1,4	14	4.42	1.87	2.61*	3.38**
492	North Argentina Offshore Front	1,2,3,4	14	3.51	1.98	0.63	0.66
493	Valdés Peninsula Front	1,2,3,4	14	2.99	1.33	1.19	1.06
494	Gulf of San Matias Front	1,2,3,4	14	3.41	1.70	-2.24	2.59*
495	Blanca Bay Front	1,2,3,4	14	4.71	2.22	1.16*	3.62**
496	South Valdés Peninsula Front	3,4	14	2.71	1.01	5.46*	-0.77
497	Autumn Valdés Peninsula Offshore Front	1	14	4.38	2.00	1.8	0.69
498	South La Plata River Front	1,2,3,4	14	2.97	1.75	6.34**	2.25**
499	North La Plata River Front	1,2,3,4	14	2.77	1.56	1.39	0.74
500	South Brazil Front	1,2,3,4	15	3.45	1.55	-5.48**	0.01
501	Santa Catarina Island Front	1,3,4	15	4.36	1.89	1.38	3.40*
502	Middle Brazil Current Front	1,2,3,4	15	4.84	1.86	3.43**	4.29**
503	Brazil Current Front	1,2,3,4	16	3.80	0.79	6.21**	1.45*
504	Vitória-Trindade Ridge Front	2,3	16	3.27	0.64	10.59**	2.12
505	North Brazil Current Front	1,2,3,4	16	4.84	0.76	2.23*	-4.04**
506	Summer North Brazil Current Front	2	16	4.44	0.61	15.74**	8.00*
507	Summer Ceara-Paraiba Offshore Front	2	16	1.94	0.40	8.81*	-6.41*
508	North Brazil-Venezuela Coastal Front	1,2,3,4	17	3.14	0.95	-5.64*	-8.57**
509	Amazon Estuary Offshore Front	1,2,3,4	17	3.20	0.71	10.97**	3.45**
510	Spring South Equatorial Current Front	1	17	2.72	0.50	-1.93	-0.37
511	Surinam Offshore Front	2,3,4	17	2.65	0.90	12.17**	2.36*
512	Tobago Coastal Front	1,4	12	1.41	0.49	10.37*	-1.16
513	Paria Peninsula Front	1,2,3,4	12	3.69	1.61	4.53**	6.88**
514	Margarita Island Front	1,2,3,4	12	3.02	1.48	0.46	4.35**
515	Golfo Triste Front	1,2,3,4	12	1.93	1.24	-3.44	1.34
516	West Venezuela Front	1,2,3,4	12	3.13	1.51	3.95**	6.52**
517	Gulf of Venezuela Front	1,2,3,4	12	4.51	2.26	1.38	3.65*
518	Lake Maracaibo Front	1,4	12	2.09	1.26	4.04	-0.49
519	West Guajira Peninsula Front	1,2,3,4	12	2.20	1.91	-0.12	3.11*
520	North Colombia Front	1,2,3,4	12	3.28	1.35	-3.83*	-1.64
521	West Caribbean Sea Coastal Front	1,2,3,4	12	2.74	0.76	-4.42*	-2.64**
522	Honduras Coastal Front	1,2,3,4	12	2.45	0.66	5.07**	-5.06**
523	Honduras Offshore Front	1,2,3,4	12	1.89	0.47	14.58**	-2.63**
524	Gulf of Honduras Front	1,2,3,4	12	4.07	1.02	4.86**	0.26
525	Guadeloupe Front	1,2,3,4	12	2.18	0.49	-5.50*	-11.11**
526	Saint Kitts and Nevis Front	1,3,4	12	1.69	0.39	3.59	-3.95**
527	Spring Anegada Passage Front	1		2.81	0.49	3.11	-3.41
528	North Puerto Rico Front	1,2,3,4	12	2.59	0.54	-6.16	-5.75**
529	South Puerto Rico Front	1,2,3	12	1.88	0.55	-18.63**	-9.83**

530	South Hispaniola Island Front	1,2,3,4	12	3.03	0.77	-4.49*	-5.50**
531	South Jamaica Front	1,2,3,4	12	2.70	0.59	1.41	-4.05**
532	Winter East Jamaica Channel Front	4	12	2.38	0.60	9.44*	0.09
533	Pedro Bank Front	1,2,4	12	2.56	0.56	3.15	-3.76**
534	West Jamaica Coastal Front	1,4	12	2.17	0.56	-2.2	-7.00**
535	Windward Passage Front	1,4	12	2.40	0.74	-4.76*	-3.71**
536	Spring South Bahamas Front	1	12	2.22	0.86	-10.58*	-0.61
537	Winter Turks and Caicos Islands Front	4	12	1.62	1.06	-15.29*	5.46*
538	East South Cuba Front	1,2,3,4	12	4.92	1.36	2.59*	1.69
539	West South Cuba Front	1,2,3,4	12	5.14	1.73	4.83**	4.91**
540	West Cuba Front	1,2,3,4	12	1.60	1.12	10.70**	2.54
541	North Cuba Front	1,2,3,4	12	3.36	1.35	13.93**	7.95**
542	Summer Nipe Bay Front	2	12	1.32	0.71	-0.74	-1.96
543	Cay Sal Bank Front	2,4	12	1.20	1.30	5.04	-1.03
544	Inagua Islands Front	1,4	12	1.90	0.64	-4.07	-8.05**
545	Outer Great Bahama Bank Front	1,2,3,4	12	4.32	1.61	7.91**	2.92*
546	Inter Great Bahama Bank Front	1,2,3,4	12	3.28	1.35	10.81**	3.12*
547	Winter Cat Island Front	4	12	2.80	0.87	18.16**	2.31
548	East Yucatan Channel Front	1,2,4	5	2.02	0.76	12.50**	-3.98**
549	Summer West Yucatan Channel Front	2	12	1.83	0.86	2	-1.9
550	Loop Current Front	1,2,3,4	5	3.22	1.35	4.66*	-0.88
551	Summer North Campeche Bank Front	2	5	4.57	1.94	4.90**	5.34*
552	Campeche Bank-Bay Coastal Front	1,2,3,4	5	4.31	1.56	5.05**	2.54*
553	Campeche Bank Offshore Front	1,2,3	5	3.97	1.76	-2.43	4.88**
554	Winter West Campeche Bank Front	4	5	3.26	0.81	14.18**	2.01
555	Spring North Campeche Bank Front	1	5	2.90	0.74	4.97	-6.20*
556	West Gulf of Mexico Front	1,2,3,4	5	4.34	1.73	4.57**	4.68**
557	North Gulf of Mexico Front	1,2,3,4	5	4.31	2.77	1.42	7.73**
558	East Gulf of Mexico Front	1,2,3,4	5	4.01	2.15	6.80**	6.98**
559	Winter Texas-Louisiana Shelf Front	4	5	7.60	5.14	-1.13	6.68**
560	Spring Texas-Louisiana Shelf Front	1	5	4.44	1.96	1.46	-0.04
561	Spring North Florida Shelf Front	1	5	3.86	2.22	1.38	3.4
562	Winter East Florida Shelf Front	4	5	3.89	3.41	5.89*	8.32**
563	South Inshore Gulf Stream Front	1,2,3,4	6	5.03	3.02	3.31**	3.39**
564	Great Bahama Bank Front	1,2,3,4	6	2.91	2.04	8.74**	5.45**
565	South Offshore Gulf Stream Front	1,2,3,4	6	4.06	1.86	5.06**	0.97
566	North Gulf Stream Front	1,2,3,4	6	5.29	5.55	1.78*	6.12**
567	South Gulf Stream Extension Front	1,2,3,4		3.51	2.85	-1.6	3.86*
568	West Blake Plateau Coastal Front	1,2,3	6	3.06	2.26	6.80**	4.45**
569	Chesapeake Bay Offshore Front	1,2,3,4	7	2.33	3.26	14.51**	5.28**
570	New York Bight Front	1,2,3,4	7	2.82	3.00	-1.08	6.42**
571	Summer Georges Bank Front	1,2,3,4	7	2.64	4.31	1.97	4.36*
572	Maine Coastal Current Front	1,2,3,4	7	3.77	2.61	2.15*	8.58**
573	Georges Bank Front	1,2,3,4	7	2.99	2.53	1.15	6.15**
574	Jordan Basin Front	1,2,3,4	7	3.31	2.44	-0.71	5.50**

575	Wilkinson Basin Front	1,2,3,4	7	2.75	1.76	-3.09	0.69
576	Outer Bay of Fundy Front	1,2,4	7	2.33	2.75	-0.47	8.33**
577	Inner Bay of Fundy Front	1,3,4	7	3.09	2.66	-1.92	2.59**
578	Nova Scotia Peninsula Coastal Front	2,3	8	3.37	2.79	6.34**	9.10**
579	Nova Scotia Peninsula Offshore Front	1,3,4	8	4.22	2.02	1.18	5.41**
580	South Shelf Break Jet Front	1,2,3,4	7	5.04	5.70	-0.62	2.27
581	North Shelf Break Jet Front	1,2,3,4	9	5.00	3.49	-3.75**	3.80*
582	North Gulf Stream Extension Front	1,2,3,4		4.47	5.35	-3.83**	3.38*
583	South Gulf of Saint Lawrence Front	1,2,3,4	8	2.84	1.76	-1.43	2.60**
584	Magdalen Islands Front	2,3	8	3.02	1.97	0.63	1.73
585	Laurentian Channel Front	1,2,3,4	9	2.16	1.51	7.44**	6.65**
586	North Gulf of Saint Lawrence Front	1,2,3,4	9	3.22	1.76	10.07**	11.95**
587	East Gulf of Saint Lawrence Front	2,3	9	3.56	2.41	-0.55	3.36**
588	Port au Port Peninsula Front	1,2	9	1.92	1.62	-9.67*	4.68*
589	Winter Cabot Strait Front	4	8	3.47	1.03	-3.3	4.91**
590	South Newfoundland Front	1,2,3	9	2.07	1.41	-3.65	3.86*
591	Winter Prince Edward Island Front	4	8	2.09	0.95	10.25*	12.80**
592	Outer Grand Banks Ring Front	1,2,3,4	9	4.38	3.57	0.78	5.48**
593	South Grand Banks Front	1,2,3,4	9	3.84	2.31	-0.77	4.09**
594	Central Grand Banks Front	1,2,4	9	3.14	1.50	-5.09*	3.1
595	North Grand Banks Front	1,2,3,4	9	3.12	1.79	2.53	4.60**
596	South Flemish Cap Front	1,2,3,4		4.88	5.31	1.5	5.17**
597	North Flemish Cap Front	1,2,3,4	9	3.65	2.84	-0.16	0.52
598	Central Flemish Cap Front	1,2,3,4	9	3.28	2.06	-4.40*	0.63
599	East Flemish Cap Front	1,2,3,4		4.18	3.46	-0.99	4.59**
600	North Newfoundland Front	1,2,3,4	9	3.17	1.92	2.45	4.63**
601	East Newfoundland Front	3,4	9	2.83	1.34	-1.39	4.05*
602	South Funk Island Bank Front	2,3,4	9	2.51	1.37	0.02	3.44**
603	Belle Isle Bank Front	2,3,4	9	3.00	1.49	0.24	5.73**
604	Labrador Coastal Front	2,3,4	9	2.83	1.62	2.77	7.40**
605	Labrador Offshore Front	2,3	9	2.53	1.59	2.51	7.22**
606	Labrador Current Front	1,2,3,4	18	5.28	2.70	3.18**	9.39**
607	Orphan Knoll Front	1,2,3,4		3.40	1.84	-5.08*	-0.69
608	East Hamilton Bank Front	1,2,3,4		2.39	1.14	-6.15*	-0.55
609	North Atlantic Current Branch Front	1,2,3,4		4.05	2.28	-0.05	7.54**
610	North Atlantic Current Front	1,2,3,4		4.47	1.50	-4.49**	2.42*
611	West Greenland Current Front	1,2,3,4	18	6.51	4.46	-0.33	7.90**
612	Central Labrador Sea Front	1,4		3.19	0.98	-0.15	4.25
613	North Labrador Sea Front	1,2,3,4	18	3.42	1.32	3.79	10.56**
614	Autumn-Winter North Labrador Sea Front	3,4		2.39	0.78	-2.59	6.33**
615	Autumn North Labrador Sea Front	3	18	4.20	1.37	-1.88	0.86
616	Winter North Labrador Sea Front	4		2.36	0.53	0	2.91
617	Baffin Island Current Front	2,3	18	4.47	2.00	1.55	7.08**
618	Frobisher Bay Offshore Front	2,3	18	3.48	1.88	-0.66	7.73*
619	Frobisher Bay Inner Front	2,3	18	2.61	1.33	0.92	10.79**

620	Cumberland Sound Coastal Front	2,3	18	3.18	2.19	3.64	10.43*
621	Cumberland Sound Offshore Front	2,3	18	2.60	1.28	1.47	11.00**
622	West Sermersooq Coastal Front	2,4	18	2.26	1.72	7.98*	7.38**
623	West Qeqqata Coastal Front	3,4	18	2.18	0.95	6.2	8.81**
624	Summer West Qeqqata Offshore Front	2	18	2.23	1.42	5.21	11.20**
625	West Qeqertalik Offshore Front	2,3,4	18	2.55	1.00	4.6	9.02**
626	Summer Disko Bay Offshore Front	2	18	3.31	3.59	-5.43	3.18
627	South Disko Bay Front	1,2,3,4	18	2.36	3.47	-15.78**	8.46**
628	North Disko Bay Front	1,2,3,4	18	2.94	3.09	11.05**	15.59**
629	West Disko Island Offshore Front	2,3	18	3.12	1.40	-6.30**	1.18
630	West Nuussuaq Peninsula Offshore Front	2,3,4	18	2.37	1.04	8.05**	6.87**
631	South Uummannaq Fjord Front	2,3	18	3.10	3.29	4.43	13.91**
632	North Uummannaq Fjord Front	2,3,4	18	2.26	2.46	12.04**	11.46**
633	Upernavik Offshore Front	2,3,4	18	2.42	1.15	-1.88	6.78**
634	Autumn Kraulshavn Offshore Front	3	18	4.24	1.17	2.26	11.83**
635	North Baffin Bay Coastal Front	2,3	18	4.05	1.90	16.18**	21.90**
636	North Baffin Bay Front	2,3	18	2.82	1.02	1.9	6.41*
637	Murchison Sound Front	2,3	18	2.61	1.25	9.47**	14.01**
638	Smith Sound Front	2,3	18	3.47	1.31	5.89	12.88**
639	Summer North Manson Icefield Front	2	18	4.13	2.00	-16.19**	-4.42
640	Summer West Baffin Bay Front	2	18	3.20	2.15	1.68	2.6
641	East Lancaster Sound Offshore Front	2,3	18	3.28	1.14	5.84	9.97**
642	North Lancaster Sound Coastal Front	2,3	18	3.29	1.44	0.46	4.07
643	South Lancaster Sound Coastal Front	2,3	18	2.86	1.15	3.13	7.95**
644	Summer East Somerset Island Front	2	18	2.64	1.11	15.32**	23.85**
645	Gulf of Boothia Front	2,3	18	1.58	0.50	22.15**	38.64**
646	Summer Gulf of Boothia Front	2	18	1.53	0.51	16.81*	39.64**
647	Barrow Strait Front	2,3	18	2.36	0.70	3.14	9.05
648	Peel Sound Front	2,3	18	1.77	0.49	6.47	17.22*
649	Summer West Griffith Island Front	2	18	2.01	0.62	3.87	15.46*
650	Wellington Channel Front	2,3	18	2.64	0.78	9.62*	12.99*
651	Summer McDougall Sound Front	2	18	2.47	0.68	1.57	10.65*
652	Autumn McDougall Sound Front	3	18	1.55	0.24	10.36	18.35*
653	Penny Strait Front	2,3	18	2.05	0.45	-2.56	7.86*
654	Summer Coburg Island Front	2	18	3.70	2.09	0.29	3.3
655	Summer East Jones Sound Front	2	18	3.39	1.26	0.72	9.39*
656	West Jones Sound Front	2,3	18	1.91	0.62	10.74**	18.08**
657	Summer South Kane Basin Front	2	66	2.39	0.69	26.66**	29.46**
658	Summer North Kane Basin Front	2	66	2.67	0.70	11.87	26.65**
659	Summer Kennedy Channel Front	2	66	2.84	0.48	13.65**	11.63**
660	Ungava Bay Coastal Front	2,3,4	63	3.12	1.42	2.96*	10.73**
661	Ungava Bay Offshore Front	2,3	63	3.88	1.49	2.49*	8.45**
662	Akpatok Island Front	2,3	63	3.09	1.40	-0.3	7.82**
663	North Hudson Strait Front	2,3	63	3.84	1.89	7.37**	12.39**
664	South Hudson Strait Front	2,3	63	3.25	1.45	2.76	7.05**

665	South Foxe Peninsula Front	2,3	63	3.14	1.45	1.16	7.38**
666	Hudson Strait-Southampton Island Front	2,3	63	4.18	2.30	0.54	5.73*
667	Digges Islands Front	2,3	63	3.56	2.17	-5.70*	4.49
668	Foxe Channel Front	2,3	63	2.81	1.26	7.41*	13.95**
669	Autumn South Foxe Basin Front	3	63	2.26	0.63	1.2	13.55**
670	Autumn Melville Peninsula Front	3	63	3.45	0.85	8.14*	18.51**
671	East Foxe Basin Front	2,3	63	2.73	1.45	2.96	11.70**
672	East Prince Charles Island Front	2,3	63	2.66	1.32	7.57*	13.97**
673	South Prince Charles Island Front	2,3	63	2.89	1.16	-0.72	10.07**
674	Autumn Bowman Bay Offshore Front	3	63	3.15	1.10	-2.67	1.59
675	West Prince Charles Island Front	3	63	3.49	1.04	5.60*	11.37**
676	North Prince Charles Island Front	2,3	63	2.17	1.23	8.05*	15.98**
677	Parry Bay Front	2,3	63	3.34	1.33	-3.89	8.42**
678	East Fury and Hecla Strait Front	2,3	63	2.31	1.24	3.55*	11.55**
679	West Southampton Island Front	2,3	63	3.68	2.40	6.93**	10.40**
680	South Southampton Island Front	2,3	63	3.29	1.56	-1.93	5.98**
681	Coats Island Front	2,3	63	3.21	1.31	-0.78	5.49*
682	Bay of Gods Mercy Front	2,3	63	3.26	2.09	7.70*	11.55**
683	West Hudson Bay Front	2,3	63	3.47	1.71	9.93**	11.87**
684	Autumn Central Hudson Bay Front	3	63	2.66	0.60	-2.36	8.05*
685	North Button Bay Coastal Front	2,3	63	3.17	1.83	6.28**	10.62**
686	Button Bay Offshore Front	2,3	63	4.18	2.36	6.66**	12.93**
687	South Hudson Bay Front	2,3	63	4.30	2.67	5.18**	14.82**
688	Autumn South Hudson Bay Front	3	63	3.71	1.31	-1.98	5.80*
689	James Bay Front	2,3	63	3.63	3.59	5.52**	16.00**
690	South James Bay Front	2,3	63	3.63	4.48	-0.41	11.45**
691	Belcher Islands Semiring Front	2,3	63	3.72	2.24	2.33	8.18**
692	Kuujjuarapik Coastal Front	2,3	63	2.70	2.01	-3.74	6.68**
693	Autumn Belcher Islands Front	3	63	2.44	1.17	-7.37*	3.46
694	Autumn West Belcher Islands Front	3	63	2.48	0.85	-5.75	7.86*
695	Summer North Belcher Islands Front	2	63	4.25	2.93	2.41	6.51*
696	West Ungava Peninsula Front	2,3	63	3.01	1.76	-0.15	5.46**
697	Autumn East Gilmour Island Front	2,3	63	2.65	1.44	4.74	6.18**
698	Autumn West Gilmour Island Front	3	63	2.95	0.73	-7.29	4.38*
699	South Irminger Current Front	1,2,3,4		3.38	0.74	-0.78	1.47
700	North Irminger Current Front	1,4		3.23	0.74	-2.03	0.3
701	East Greenland Coastal Current Front	2,3	19	3.32	1.89	0.75	11.79**
702	East Greenland Current Front	1,2,3,4	19	6.64	6.14	0.56	4.94**
703	Sermilik Deep Front	1,2,3,4	19	6.14	3.93	-3.67*	-0.06
704	Greenland-Iceland Rise Front	1,2,3,4	19	2.75	1.70	1.57	10.60**
705	Denmark Strait-Kolbeinsey Ridge Front	1,2,3,4	19	3.05	1.15	10.73**	14.59**
706	Greenland Abyssal Plain Front	1,2,3,4	19	3.14	0.82	3.32	11.44**
707	Spring Greenland Abyssal Plain Front	1	19	2.21	0.38	4.95	19.28**
708	Autumn Greenland Abyssal Plain Front	3	19	2.62	0.67	-3.85	15.75**
709	North East Greenland Current Front	1,2,3,4	19	4.37	1.55	4.28*	17.37**

710	Jan Mayen Current Front	1,2,3,4	19	5.71	1.90	-1.95**	0.51
711	North Mohns Ridge Front	1,2,3,4	19	4.90	2.16	-1.33	1.64
712	East Greenland Ridge Front	1,2,3,4	19	3.59	1.13	-3.55*	5.53*
713	South East Greenland Ridge Front	1,3,4	19	3.17	0.84	-2.34	10.47**
714	North Greenland Abyssal Plain Front	1,4	19	3.54	0.65	-0.75	17.71**
715	Eggvin Shoal Front	3,4	19	3.81	1.02	-2.31	2.97
716	Hovgaard Ridge Front	1,3,4	19	3.03	1.14	-4.79	9.07**
717	Fram Strait Front	1,2,3,4	19	5.36	2.69	3.83**	11.32**
718	Summer Holm Land Front	2	19	3.94	0.79	5.97*	8.97*
719	Summer Hovgaard Island Offshore Front	2	19	3.34	0.66	-12.71**	3.59
720	Summer Hochstetter Bay Front	2	19	2.63	1.23	-0.26	5.33
721	North Iceland Coastal Front	1,2,3,4	59	3.12	2.04	5.91**	1.81*
722	Faza Bay Front	1,4	59	3.99	1.96	4.36**	5.60**
723	West Iceland Offshore Front	2,3	59	2.97	0.68	2.15	2
724	Reykjanes Ridge Front	1,2,3,4	59	3.45	0.71	1.45	3.37*
725	Reykjanes Ridge Branch Front	1,4	59	3.74	0.56	4.68	5.15**
726	South Icelandic Current Front	1,2,3,4	59	5.57	1.56	1.31*	9.26**
727	East Icelandic Current Front	1,2,3,4	21	5.88	2.56	-0.43	4.05*
728	Denmark Strait Front	1,2,3,4	59	6.90	3.64	0.23	5.88**
729	North Icelandic Current Front	1,2,3,4	59	5.81	1.95	-1.49	3.13*
730	North Iceland Offshore Front	2,3	59	4.26	2.02	1.13	-1.18
731	South Iceland Offshore Front	1,2,3,4		2.63	0.50	-1.48	4.84
732	East Reykjanes Ridge Front	2,3,4		2.34	0.48	4.5	4.52*
733	South Jan Mayen Ridge Front	1,2,3,4	59	3.69	1.21	-0.19	3.11*
734	North Icelandic Current Branch Front	1,2,3,4	59	3.62	1.00	-1.12	6.44**
735	West Jan Mayen Ridge Front	1,4	59	2.96	0.70	-1.84	4.64*
736	Central Icelandic Plateau Front	1,2,3,4	59	2.35	0.81	7.08	8.22**
737	Iceland-Faroe Ridge Front	1,2,3,4	59	3.78	1.04	-3.34**	-1.4
738	Lousy Bank Front	1,2,3,4		2.73	0.45	-9.28**	-0.84
739	Faroe Islands Ring Front	1,2,3,4	60	4.19	1.14	4.78**	5.51**
740	West Faroe Islands Front	1,2,3,4	60	3.67	0.73	-3.25*	0.86
741	East Faroe Islands Front	1,2,3,4	22	4.01	1.21	-4.07*	1.38
742	Faroe-Shetland Channel Front	1,2,3,4	60	5.06	1.04	-2.50*	1.48
743	Treitel Ridge Front	1,2,3,4	21	3.68	1.37	-5.88*	2.32
744	West Vøring Plateau Front	1,4	21	3.93	0.88	2.09	2.65
745	Aegir Ridge Front	1,2,3,4	21	2.71	0.82	-0.69	1.03
746	Jan Mayen Fracture Zone Front	1,2,3,4	21	3.91	0.89	-1.87	4.23**
747	Dumshaf Abyssal Plain Front	1,2,3,4	21	3.73	0.77	-4.80**	1.08
748	South Jan Mayen Fracture Zone Front	1,2,3,4	21	2.76	0.79	10.68**	5.65**
749	South Norway Offshore Front	1,2,3,4	21	4.02	1.28	-3.38**	1.99
750	South Norway Coastal Front	1,2,3,4	21	3.14	1.34	1.45	6.23**
751	West Norway Coastal Front	1,2,3,4	21	3.51	1.35	3.02*	9.06**
752	South Trøndelag Platform Front	1,4	21	2.70	0.58	1.23	-0.77
753	North Trøndelag Platform Front	2,3,4	21	2.53	0.71	1.89	3.88*
754	East Vøring Plateau Front	1,2,3,4	21	3.93	0.74	-0.67	1.95*

755	Central Vøring Plateau Front	1,2,3,4	21	4.00	0.74	-5.16**	1.07
756	Winter-Spring Central Vøring Plateau Front	1,4	21	3.27	0.54	-7.26*	-0.19
757	Summer Central Vøring Plateau Front	2	21	4.10	0.91	-1.51	4.92*
758	Vestfjorden Front	1,2,3,4	21	3.36	1.04	0.53	4.88**
759	Summer Lofoten Archipelago Offshore Front	2	21	3.69	0.98	0.38	1.27
760	Knipovich Ridge-Bear Island Front	1,2,3	21	3.45	0.86	-1.63	0.88
761	West Svalbard Archipelago Front	2,3	19	2.99	0.70	9.04*	5.58*
762	Southwest Bear Island Front	1,2,3,4	21	3.33	0.86	-2.81	0.39
763	Lofoten Archipelago Coastal Front	1,3,4	20	4.63	1.42	3.64**	5.77**
764	North Norway Coastal Front	1,2,3,4	20	3.21	1.08	1.31	3.26**
765	North Norway Offshore Front	1,2,3,4	20	2.86	0.63	-5.47**	-0.4
766	North Cape Front	1,2,3,4	20	2.03	1.03	6.26**	6.09**
767	South Barents Sea Front	1,2,3,4	20	4.97	1.11	-2.01*	1.76
768	Central Barents Sea Front	1,2,3,4	20	5.26	1.31	-1.22	4.21**
769	South Central Bank Front	1,2,3,4	20	3.53	1.20	1.78	3.62**
770	North Sentralbanken Front	1,2,3,4	20	2.90	0.81	13.21**	15.60**
771	North Kola Peninsula Coastal Front	1,2,3,4	20	4.72	1.38	7.05**	5.06**
772	North Kola Peninsula Offshore Front	2,3,4	20	3.31	0.92	-5.49**	-0.35
773	Kola Peninsula-Pechora Sea Front	1,2,3,4	20	3.41	0.99	2.62*	4.21**
774	North Murman Rise Front	1,2,3,4	20	4.30	1.17	2.46	2.73*
775	Kanin Peninsula-Pechora Sea Front	1,2,3,4	20	3.14	1.22	6.81*	8.36**
776	East Kola Peninsula Front	1,2,3,4	20	3.54	1.53	8.67**	7.39**
777	White Sea Front	1,2,3,4	20	2.81	1.70	4.90**	6.46**
778	Dvinsk Bay Front	2,3,4	20	2.58	2.13	5.97**	8.99**
779	Onega Bay Front	2,3,4	20	3.81	3.01	9.41**	13.09**
780	South Kanin Bank Front	1,2,3,4	20	3.70	1.20	1.16	4.53*
781	South Pechora Sea Coastal Front	1,2,3,4	20	3.22	1.72	5.58**	10.78**
782	South Kolguyev Island Front	2,3	20	3.21	1.59	-1.24	3.29*
783	Kanin Peninsula Front	2,3,4	20	3.27	1.53	-0.38	5.91**
784	Goose Bank-Novaya Zemlya Front	1,2,3,4	20	3.73	1.34	6.90**	9.96**
785	West Goose Bank Front	1,2,3,4	20	3.63	0.95	2.96	2.21
786	North Bear Island Front	1,2,3,4	20	3.78	2.41	-3.92*	0.13
787	South Bear Island Front	1,2,3,4	20	6.42	3.47	-3.21**	3.29*
788	Storfjorden Trough Front	1,2,3,4	20	3.02	1.33	17.00**	23.45**
789	South Svalbard Archipelago Front	1,2,3,4	20	5.28	2.72	3.83**	9.17**
790	Spitsbergenbanken-Novaya Zemlya Bank Front	1,2,3,4	20	6.07	1.96	2.54	12.42**
791	North Novaya Zemlya Front	1,2,3,4	20	3.24	0.90	9.81**	10.33**
792	North Novaya Zemlya Bank Front	1,3,4	20	2.53	0.71	32.25**	30.20**
793	North North-Eastern Basin Front	1,2,3,4	20	3.68	0.86	21.45**	19.23**
794	Svalbard Archipelago-Franz Josef Land Front	2,3	20	3.30	0.92	22.68**	26.53**
795	East Edgeøya Front	2,3,4	20	3.62	1.33	12.82**	18.06**
796	Kong Karls Land Front	2,3	20	3.80	0.97	4.4	13.06**
797	North Great Bank Front	2,3	20	3.80	0.89	5.88*	5.40*
798	East Storbanken Front	1,2,3,4	20	3.05	0.65	16.07**	19.57**
799	North Svalbard Archipelago Front	2,3	20	4.74	1.96	1.37	0.91

800	Autumn North Svalbard Archipelago Front	3	20	2.54	0.50	5.49	20.44**
801	North Franz Josef Land Front	2,3	20	1.88	0.30	43.84**	47.03**
802	East Kvitøya Front	2,3	20	1.99	0.40	17.33*	31.12**
803	Russian Arctic National Park Offshore Front	2,3	58	3.77	0.89	17.47**	19.42**
804	Cape Flissingsky Front	2,3	58	3.62	1.19	15.48**	16.18**
805	East Novaya Zemlya Coastal Front	2,3	58	2.89	1.31	15.70**	14.72**
806	Autumn East Severny Island Offshore Front	3	58	3.88	1.07	5.95	13.45**
807	Autumn East Yuzhny Island Offshore Front	3	58	4.07	1.21	3.14	12.54**
808	South Kara Sea Offshore Front	2,3	58	3.13	1.38	4.14*	7.17**
809	South Kara Sea Coastal Front	2,3	58	3.18	1.88	8.13**	10.09**
810	North Yamal Peninsula Front	2,3	58	2.81	1.51	7.06**	13.02**
811	Summer Central Kara Sea Front	2	58	3.63	2.13	6.13*	-0.2
812	Summer Gyda Peninsula Front	2	58	4.32	2.48	-0.94	4.27
813	Summer Sverdrup Island Front	2	58	3.62	2.20	9.46**	9.87**
814	Summer Izvestiy TSIK Islands Front	2	58	3.03	1.55	17.82**	20.62**
815	North Uyedineniya Island Front	2,3	58	2.53	0.71	19.66**	34.69**
816	Autumn Wiese Island Front	3	58	1.98	0.40	15.17*	40.58**
817	North Ushakov Island Front	2,3	58	1.54	0.25	66.15**	67.74**
818	Autumn West Severnaya Zemlya Front	3	58	1.80	0.39	54.88**	51.10**
819	North Krasnoyarsk Krai Front	2,3	58	2.47	0.88	14.00**	27.31**
820	Vilkitsky Strait Front	2,3	57	2.38	0.71	22.02**	33.66**
821	Summer North Severnaya Zemlya Front	2	57	2.06	0.40	18.81*	30.20*
822	Taymyr Peninsula Front	2,3	57	2.50	0.82	22.26**	29.00**
823	Olenekskiy Bay Front	2,3	57	2.35	1.36	17.43**	21.27**
824	Lena Delta Coastal Front	2,3	57	3.10	1.82	9.31**	20.07**
825	Summer Laptev Sea Rift Front	2	57	3.43	1.93	1.79	3.62
826	Summer Lena Delta Offshore Front	2	57	4.03	2.46	-0.86	8.11**
827	Autumn Laptev Sea Front	2	57	2.20	0.88	31.89**	36.04**
828	West New Siberian Islands Front	2	57	3.87	1.70	9.12*	16.86**
829	Sannikov Strait Front	2,3	57	2.14	0.88	9.51*	18.78**
830	Yana Bay Front	2,3	57	2.27	1.31	12.42**	21.68**
831	Summer Yana Bay Offshore Front	2	57	3.02	1.75	2.56	14.21*
832	Summer East Lyakhovsky Islands Front	2	57	1.89	1.19	13.94**	19.39**
833	Kotelny Island Front	2,3	57	2.61	0.99	20.17**	32.24**
834	Summer North Anzhu Islands Offshore Front	2	57	3.53	1.22	2.37	16.07**
835	Summer North Anzhu Islands Coastal Front	2	57	3.58	1.38	11.85**	24.02**
836	Autumn South Anzhu Islands Coastal Front	3	57	1.90	0.46	9.21*	14.21**
837	East Siberian Sea Coastal Front	2,3	56	2.89	1.41	10.32**	12.84**
838	Kolyma Gulf Front	2,3	56	2.64	1.57	10.84**	15.95**
839	Autumn West Wrangel Island Front	3	56	2.34	0.63	30.26**	42.43**
840	Norwegian Coastal Current Front	1,2,3,4	22	3.72	1.51	0.43	2.90*
841	Forty Mile Ground Front	1,3,4	22	3.23	0.70	-1.34	-0.67
842	Summer Shetland Islands Front	2	22	3.85	1.30	10.53**	10.31**
843	West Scotland Front	1,2,3,4	22	3.46	0.91	1.24	3.14*
844	Fair Isle Strom Front	3,4	22	2.66	0.64	2.93	1.87

845	Winter Orkney Islands Front	4	22	1.88	0.68	8.89*	7.54**
846	Moray Firth Front	1,2,3,4	22	3.25	0.89	4.16**	4.71**
847	West North Sea Coastal Front	1,2,3,4	22	3.49	1.39	4.33**	4.65**
848	Summer Firth of Forth Offshore Front	2	22	3.60	1.32	-1.35	2.33
849	Fladen Ground Front	2,3,4	22	2.95	0.69	-1.43	0.22
850	Devils Hole Front	2,3,4	22	2.66	0.82	0.04	1.67
851	Dogger Bank Ring Front	1,2,3,4	22	3.04	0.92	0.69	1.28
852	Central Dogger Bank Ring Front	2,3	22	2.99	1.00	1.42	-0.09
853	Flamborough Head Front	1,2,3	22	3.29	1.36	6.38*	2.37
854	West Southern Bight Front	1,2,3,4	22	3.62	1.70	1.17	3.18**
855	East Southern Bight Front	1,2,3,4	22	4.77	2.24	2.23*	3.48**
856	German Bight Front	1,2,3,4	22	5.14	2.44	2.42**	6.92**
857	North Frisian Islands Front	1,2	22	3.74	2.08	2.24	1.66
858	North Skagerrak Strait Front	1,2,3,4	22	3.16	1.94	0.13	2.60*
859	Fisher Bank Front	3,4	22	3.57	1.23	0.09	4.90**
860	Autumn East Dogger Bank Front	3	22	3.79	1.23	2.54	3.81*
861	Winter Eigersund Bank Front	4	22	3.37	0.86	-10.60*	-1.45
862	East Kattegat Strait Front	2,3,4	22	2.34	1.54	2.47	2.41*
863	Autumn West Kattegat Strait Front	3	22	2.10	1.05	17.09**	7.61**
864	South Baltic Sea Front	1,2,3,4	23	4.53	2.08	0.55	2.60*
865	Hanö Bay Front	1,2,3,4	23	3.11	1.49	7.12**	4.92**
866	Winter Gotland Front	4	23	3.14	0.96	-5.41*	6.32**
867	Autumn Gotland Deep Front	3	23	3.12	1.33	-11.47*	-5.93
868	Autumn South Blatic Sea Front	3	23	3.60	1.52	3.98	2.88
869	East Gotland Deep Coastal Front	1,2,3,4	23	3.44	2.33	8.60**	0.11
870	Gulf of Riga Front	1,2,3,4	23	2.73	2.43	4.70*	-0.52
871	South Gulf of Finland Front	1,2,3,4	23	2.72	2.21	1.91	-0.59
872	North Gulf of Finland Front	1,2,3,4	23	3.54	2.84	2.18	3.90*
873	Central Baltic Sea Front	1,2,3,4	23	3.88	1.96	7.44**	4.42**
874	Bothnian Sea Ring Front	1,2,3,4	23	3.36	1.87	5.42**	3.94*
875	South Gulf of Bothnia Front	1,2,3,4	23	3.46	2.17	9.39**	9.34**
876	North Gulf of Bothnia Front	1,2,3,4	23	3.48	2.18	11.26**	12.15**
877	Rosemary Bank Front	1,2,3,4		3.34	0.60	-2.6	-0.18
878	West Rosemary Bank Front	1,2,3,4		3.65	0.57	-6.86**	-1.26
879	Rockall Bank Front	1,2,3,4		4.67	0.84	-2.03*	-0.13
880	East Rockall Trough Front	1,4	24	3.64	0.82	4.53	2.56
881	Malin Coastal Front	1,2,4	24	3.31	1.07	-4.24*	0.77
882	North Ireland Coastal Front	1,2,3,4	24	3.85	1.06	-1.4	1.24
883	Donegal Bay Front	1,2,3,4	24	2.79	1.04	-9.37**	2.60*
884	Galway Bay Front	1,2,3,4	24	2.68	1.01	-3.61*	3.30**
885	South Ireland Coastal Front	1,2,3,4	24	3.89	1.26	4.57**	6.11**
886	Porcupine Bank Ring Front	1,2,3,4	24	2.93	0.59	0.31	1.19
887	South Rockall Plateau Margin Front	1,2,3,4		3.64	0.73	-0.87	5.81**
888	North Irish Sea Front	1,2,3,4	24	3.86	1.83	2.57*	3.78**
889	Autumn Isle of Man Front	3	24	2.71	1.12	-4.16	0.51

890	Cardigan Bay Front	1,2,3,4	24	3.31	2.01	4.22**	2.67*
891	Bristol Channel Front	1,2,3,4	24	3.63	1.59	3.91**	2.71*
892	Summer Trevose Head Front	2	24	1.40	1.79	-4.7	3.78*
893	North English Channel Front	1,2,3,4	24	3.99	1.64	5.24**	4.05**
894	South English Channel Front	1,2,3,4	24	5.19	2.07	2.01*	4.83**
895	Channel Islands Front	1,2,3,4	24	2.99	1.65	-1.51	3.25*
896	West Channel Islands Front	1,3	24	3.42	1.26	-3.26	-1.08
897	Winter South Ireland Offshore Front	4	24	3.81	0.99	4.04	4.30**
898	Autumn South Celtic Sea Front	3	24	3.67	0.93	-2.04	1.41
899	Iroise Sea Offshore Front	1,2,3	24	3.40	1.64	1.89	6.20**
900	South Celtic Sea Shelf Front	1,4	24	4.92	0.85	4.94*	5.12**
901	Celtic Sea Slope Front	1,2,3,4	24	3.79	0.85	-1.27	2.79**
902	Porcupine Abyssal Plain Front	1,2,3,4		3.18	0.64	-2.42	3.37*
903	South Celtic Sea Front	3,4	24	3.11	0.57	-1.98	0.75
904	West Cantabrian Sea Front	1,3,4		2.79	0.57	-2.52	-0.1
905	North Bay of Biscay Coastal Front	2,3,4	24	4.69	2.23	2.99*	7.34**
906	Spring North Bay of Biscay Offshore Front	1	24	3.56	1.29	-2.55	-1.05
907	Spring Rochebonne Shoal Front	2,3	24	2.64	1.34	-2.31	2.1
908	Autumn North Landes Plateau Front	3	24	2.95	0.91	3.48	1.2
909	South Bay of Biscay Coastal Front	1,2,3,4	25	3.87	1.28	0.77	5.73**
910	South Bay of Biscay Offshore Front	4	25	3.88	0.69	-9.62*	-7.03*
911	West Iberian Peninsula Front	1,2,3,4	25	4.05	1.69	1.44	9.28**
912	Galicia Bank Front	1,2,4	25	3.23	0.84	-1.96	1.75
913	Estremadura Spur Front	1,4	25	3.42	0.95	1.84	5.15*
914	Gorringe Bank Front	1,2,3,4	25	3.15	0.94	1.56	4.76**
915	North Gulf of Cádiz Coastal Front	1,2,3,4	25	4.93	2.10	4.29**	8.55**
916	West Alboran Sea Ring Front	1,2,3,4	26	3.34	1.75	2.08	4.95**
917	Central Alboran Sea Ring Front	1,2,3,4	26	3.29	1.54	0.77	2.63*
918	East Alboran Sea Ring Front	1,2,3,4	26	3.06	1.25	-3.68**	-1.14
919	Southwest Spain Coastal Front	1,2,3,4	26	2.92	1.13	-4.46*	-1.58
920	Algeria Coastal Front	1,2,3,4	26	3.44	1.07	-4.90*	-1.94
921	Algeria-Tunisia Coastal Front	1,2,3,4	26	2.63	0.96	-12.38**	-4.42**
922	Balearic Islands Front	1,4	26	3.87	1.02	-2.63	-3.73*
923	West Balearic Sea Front	1,2,4	26	3.03	1.03	4.50**	2.44*
924	Summer South Balearic Islands Front	2	26	2.46	0.99	9.77	-2.8
925	North Balearic Sea Front	1,2,3,4	26	3.83	1.48	2.26*	3.78**
926	Gulf of Lion Front	1,2,3,4	26	3.73	1.64	3.58*	7.46**
927	Autumn North Balearic Sea Front	3	26	5.10	2.36	1.47	3.95*
928	Winter North Balearic Sea Front	4	26	4.15	1.04	4.59	2.1
929	Gulf of Lion-Ligurian Sea Front	1,2,3,4	26	4.06	1.45	-0.53	-0.52
930	West Corsica Island Offshore Front	3,4	26	4.39	1.46	-5.75**	-7.54**
931	West Corsica Island Coastal Front	1,2	26	2.72	1.34	-7.75**	-5.24**
932	East Ligurian Sea Coastal Front	1,2,3,4	26	2.89	1.29	2	3.57**
933	North Tyrrhenian Sea Coastal Front	1,2,4	26	2.37	1.01	-3.05*	3.04**
934	North Corsica Island Coastal Front	3,4	26	3.77	1.23	-5.16*	-4.98*

935	North Tyrrhenian Sea Ring Front	3,4	26	4.51	1.54	-2.75	1.27
936	East Corsica Island Front	1,2	26	2.45	1.36	-1.02	-2.3
937	Winter North Tyrrhenian Sea Offshore Front	4	26	2.35	0.70	-14.15*	0.96
938	East Sardinia Island Coastal Front	2,4	26	3.04	1.20	5.62*	2.93*
939	West Sardinia Island Front	1,2,3,4	26	2.42	1.05	2.47	2.77*
940	East Sardinia Island Offshore Front	2,3	26	2.35	1.03	11.82**	0.5
941	Summer Pontine Islands Offshore Front	2	26	2.79	1.09	9.15*	-1.22
942	Gulf of Salerno Offshore Front	2,3	26	2.91	1.09	4.31	-1.18
943	Autumn Aeolian Islands Front	3	26	2.18	0.99	-9.12	-1.28
944	Spring North Sicily Coastal Front	1	26	1.83	0.91	-27.68**	-6.70**
945	Winter North Sicily Offshore Front	4	26	2.58	0.61	-23.45**	-12.60**
946	Aegadian Islands Front	2,3	26	2.45	1.68	9.31*	10.52**
947	Winter South Tyrrhenian Sea Front	4	26	3.63	0.79	2.42	-1.93
948	North Tunisia Front	1,2,3,4	26	3.41	1.06	-3.27*	-2.73*
949	Autumn Strait of Sicily Front	3	26	4.75	1.70	-0.87	2.81
950	South Sicily Front	1,2,3,4	26	2.94	1.25	5.22*	5.27**
951	Gulf of Gabes Front	1,2,3,4	26	5.02	2.27	6.22**	7.70**
952	Gulf of Gabes Offshore Front	1,4	26	3.62	0.95	8.47**	4.97*
953	Malta Front	1,2,3,4	26	3.14	0.97	3.05	2.48
954	Gulf of Sidra Coastal Front	1,2,3,4	26	3.65	0.97	6.48**	1.47
955	Winter East Catania Offshore Front	4	26	3.00	0.83	0.87	-2.46
956	West Ionian Sea Front	1,2,3,4	26	2.74	1.09	-2.49	-2.46
957	Central Adriatic Sea Front	3,4	26	3.94	1.54	-4.16*	3.03*
958	Winter East Ionian Sea Front	4	26	3.38	1.06	-1.82	1.47
959	East Adriatic Sea Front	1,2,3,4	26	3.91	1.72	3.85**	7.61**
960	West Adriatic Sea Front	1,2,3,4	26	3.88	1.83	6.80**	11.92**
961	Winter West Peloponnese Front	4	26	3.00	0.91	3.72	-2.8
962	Winter Southwest Peloponnese Front	4	26	2.53	0.81	9.68*	-1.55
963	Winter Libya Front	4	26	4.08	1.00	-0.65	-4.22*
964	Crete Island Offshore Front	3,4	26	3.29	1.23	-7.04*	-5.70**
965	Crete Island Front	1,3,4	26	2.31	1.03	-5.12	-6.22*
966	Libyan-Egypt Front	1,2,3	26	2.69	0.82	-11.30**	-9.63**
967	South Levantine Sea Front	1,2,3,4	26	5.84	1.75	-0.51	0.79
968	North Levantine Sea Front	1,2,3,4	26	3.54	1.15	-9.81**	-8.27**
969	Iskenderun Bay Front	1,2,3,4	26	3.19	1.10	-3.47**	-1.14
970	East Herodotus Basin Front	2,3,4	26	3.31	0.94	-1.45	-3.61*
971	West Levantine Sea Ring Front	1,2,3,4	26	3.94	1.54	-0.56	-1.39
972	South Cyprus Front	3,4	26	3.53	1.12	4.08	-0.45
973	Winter Southeast Crete Island Front	4	26	3.32	1.43	-6.59	-6.88*
974	Northwest Egypt Offshore Front	3,4	26	3.01	0.97	-2.43	-6.24*
975	North Crete Island Coastal Front	2,3	26	2.58	1.30	-12.56**	-7.34**
976	Sea of Crete Front	1,3,4	26	2.45	1.08	-4.24*	-4.46**
977	North Cyclades Front	1,4	26	2.64	1.27	-2.89	0.63
978	Summer Sea of Crete Front	2	26	2.39	1.38	-0.97	0.28
979	Autumn North Sea of Crete Front	3	26	2.68	1.52	2.69	5.91*

980	East Peloponnese Peninsula Coastal Front	2,3	26	2.46	1.32	-4.64	-4.86**
981	Central Aegean Sea Front	3,4	26	2.43	1.45	-1.06	1.02
982	North Aegean Sea Front	1,2,3,4	26	3.03	1.82	0.78	1.47*
983	Thermaic Gulf Front	1,4	26	2.25	1.37	8.52	7.14*
984	Thracian Sea Front	1,4	26	3.84	1.86	1.94	5.41**
985	Sea of Marmara Front	1,2,3	26	1.82	1.49	-0.53	-3.90*
986	West Black Sea Front	1,2,3,4	62	3.25	1.76	1.65*	4.72**
987	Spring West Black Sea Front	1	62	4.33	1.96	2.54	3.44*
988	Odesa Shelf Front	1,2,3,4	62	3.67	2.46	0.98	7.10**
989	Winter West Crimean Peninsula Front	4	62	2.70	2.81	-5.44*	7.73**
990	North Black Sea Front	1,2,3,4	62	2.97	1.33	-2.5	-0.02
991	South Black Sea Coastal Front	1,2	62	2.56	1.28	-9.60**	-6.19**
992	Winter South Black Sea Offshore Front	4	62	3.74	1.04	-8.03*	-4.4
993	Southeast Black Sea Offshore Front	1,2	62	2.55	1.18	8.05**	-2.04
994	Southwest Black Sea Offshore Front	1,3,4	62	2.95	1.06	1.64	1.49
995	Autumn Central Black Sea Front	3	62	3.64	1.72	8.55	8.42*
996	Sea of Azov Front	1,2,3,4	62	3.30	2.12	9.97**	2.06*
997	South Strait of Gibraltar Front	1,2,3,4	27	5.31	2.22	5.65**	10.49**
998	West Strait of Gibraltar Offshore Front	1,3,4	27	2.53	0.74	0.81	-2.46*
999	West Azores Archipelago Front	1,2,3,4		3.47	0.83	6.67**	2.08
1000	Azores Archipelago Front	1,2,3,4		3.28	0.87	6.09**	2.80*
1001	East Azores Archipelago Front	1,2,3,4		3.32	0.83	1.81	2.28*
1002	South Horseshoe Abyssal Plain Front	1,4		3.65	0.76	7.00*	7.44**
1003	Madeira Archipelago Front	1,3,4		2.78	0.61	7.07*	3.67*
1004	Summer Madeira Archipelago Front	2		2.94	0.88	-3.32	1.85
1005	West Canary Islands Front	1,4		2.88	0.63	6.49	-0.42
1006	East Canary Islands Front	1,2,3,4	27	2.12	0.95	0.58	-0.98
1007	Morocco Offshore Front	1,2,3,4	27	3.89	1.62	2.54*	6.72**
1008	Western Sahara Slope Front	1,2,3,4	27	5.37	2.52	2.05**	9.98**
1009	West Bay of Arguin Offshore Front	1,2,3,4	27	2.99	2.29	-0.75	8.57**
1010	Bay of Arguin Front	1,2,3,4	27	5.52	4.18	2.24*	6.87**
1011	West Mauritania Front	1,2,3,4	27	4.57	2.63	3.04*	6.12**
1012	Senegal-Guinea Offshore Front	1,4	28	3.47	2.31	3.23**	6.97**
1013	North Cape Verde Front	1,2,3,4		2.78	0.86	2.39	2.72**
1014	South Cape Verde Front	1,2,3,4		3.20	1.04	0.04	2.14*
1015	Bissagos Islands Front	1,2,3,4	28	4.15	2.04	-2.79	3.11*
1016	North Gulf of Guinea Front	1,2,3,4	28	3.36	0.95	-0.26	3.96**
1017	North Gulf of Guinea Offshore Front	2,3	28	2.90	0.83	3.31	4.35**
1018	South Gulf of Guinea Front	1,2,3,4	28	2.65	0.98	-9.47**	-1.78
1019	Spring Atlantic Northern South Equatorial Current Front	3	28	3.44	0.81	-2.67	-1.97
1020	Atlantic Central South Equatorial Current Front	1,2,3,4		3.11	0.84	2.38*	3.22**
1021	Sao Tome and Principe Front	2,3	28	2.79	0.93	-4.68*	-3.15*
1022	Angola-Namibia Front	1,2,3,4	29	4.71	2.29	4.99**	5.88**
1023	Winter Angola Offshore Front	2	29	3.61	1.51	-4.87	2.63

1024	Namibia Front	1,2,3,4	29	5.52	3.43	4.74**	9.25**
1025	North Benguela Current Front	3,4	29	4.40	2.29	-4.18*	0.81
1026	Spring-Summer North Benguela Current Front	1,2	29	3.86	2.16	2.33	1.52
1027	South Benguela Current Front	1,2,3,4	29	3.49	1.82	4.67**	5.10**
1028	West Cape Agulhas Front	1,2,3,4	29	4.75	3.04	1.26*	8.52**
1029	Cape Agulhas Coastal Front	1,2,3,4	30	2.52	1.70	4.25**	7.41**
1030	North Agulhas Current Front	1,2,3,4	30	5.97	3.88	1.99**	6.26**
1031	South Agulhas Current Front	1,2,3,4	30	4.68	3.19	3.36**	3.63**
1032	Agulhas Current Offshore Front	1,2,3,4	30	4.17	2.31	2.51**	2.33**
1033	Agulhas Return Current Front	1,2,3,4		3.77	3.18	-0.23	3.90**
1034	Maputo Coastal Front	1,2,3,4	30	3.70	1.28	3.57**	3.94**
1035	Mozambique Front	1,2,3,4	30	4.07	1.20	4.19**	2.31*
1036	Mozambique-Tanzania Front	1,2,3,4	30	3.44	0.90	1.78*	0.39
1037	West Madagascar Coastal Front	1,2,3,4	30	4.57	1.21	3.77**	2.35*
1038	South Madagascar Offshore Front	1,2,3,4	30	3.90	1.35	1.65	-0.4
1039	Ambovombe Coastal Front	3,4	30	2.46	1.27	1.73	2.36
1040	East Madagascar Front	1,2,3,4	30	4.04	1.08	9.00**	6.28**
1041	North East Madagascar Current Front	1,2,3	30	2.81	0.80	1.05	-3.58*
1042	South East Madagascar Current Front	1,2,3,4	30	3.39	1.35	3.46*	4.51*
1043	Reunion Front	1,2,3,4		3.15	0.68	3.46	-1.85
1044	North Madagascar Front	1,2,3,4	30	3.98	0.99	2.26	-0.7
1045	Seychelles Outer Islands Front	1,2,3		2.32	0.57	11.20**	2.5
1046	Spring Comorin Front	3	30	2.27	0.70	8.99*	-4.85*
1047	Tanzania-Kenya Offshore Front	1,2,3,4	31	2.42	0.70	9.92**	-0.65
1048	Zanzibar Front	1,2,3,4	31	2.63	0.86	0.18	2.06
1049	South Somalia Offshore Front	1,2,3,4	31	2.20	0.56	5.37*	-4.32**
1050	Kenya Coastal Front	1,2,3,4	31	2.44	0.92	-1.03	0.18
1051	South Somalia Coastal Front	1,4	31	2.53	0.61	1.02	-4.37**
1052	Somalia Current Front	1,2,3,4	31	3.34	1.29	3.41**	11.28**
1053	Cape Guardafui Front	1,2,3	32	3.26	2.71	1.43	7.72**
1054	West Socotra Front	1,2	32	2.12	1.32	1.64	4.77**
1055	East Socotra Front	1,2,3	32	2.38	1.00	4.33*	3.05*
1056	South Gulf of Aden Front	1,2,3,4	32	3.14	1.35	0.19	1.83*
1057	West Bab-el-Mandeb Strait Front	1,3,4	33	3.40	1.49	0.63	1.41*
1058	East Bab-el-Mandeb Strait Front	1,2,3,4	33	4.04	1.48	1.26	1.98*
1059	East Red Sea Front	1,2,3,4	33	3.50	1.26	-0.85	1.12
1060	West Red Sea Front	1,2,3,4	33	3.26	1.22	-1.23	-0.92*
1061	Winter Suakin Archipelago Front	4	33	4.20	1.22	-1.37	2.26
1062	North Suakin Archipelago Front	1,2,3,4	33	4.00	1.31	2.02	0.83
1063	Gulf of Suez Front	1,2,3,4	33	4.83	2.22	11.30**	6.34**
1064	North Gulf of Aden Front	1,2,3,4	32	3.15	1.23	2.08*	5.23**
1065	Oman Coastal Front	1,2,3,4	32	3.56	1.78	1.82*	9.49**
1066	Summer Ras al Hadd Front	2	32	4.63	3.53	-7.94*	2.33
1067	Autumn Gulf of Oman Front	3	32	3.37	1.49	-1.25	-0.35
1068	South Gulf of Oman Front	1,2,3,4	32	3.09	1.25	-0.31	1.95

1069	East United Arab Emirates Coastal Front	1,2,3,4	32	3.93	1.61	-3.78*	-0.37
1070	West United Arab Emirates Coastal Front	1,2,3,4	32	3.34	1.43	-1.76	-1.11
1071	United Arab Emirates Offshore Front	2,3,4	32	3.59	1.43	-1.3	0.24
1072	West Persian Gulf Front	1,2,3,4	32	4.49	2.10	1.46*	3.86**
1073	North Persian Gulf Front	1,2,3,4	32	3.91	2.06	3.36**	6.06**
1074	Bushehr Offshore Front	1,4	32	3.17	1.52	-3.37	-0.26
1075	East Persian Gulf Offshore Front	1,2	32	2.63	1.17	-2.77	-6.50**
1076	East Persian Gulf Coastal Front	1,2,3,4	32	2.59	1.39	-2.78*	2.67*
1077	Autumn East Persian Gulf Offshore Front	3	32	3.11	1.30	7.47*	8.08**
1078	North Gulf of Oman Front	1,2,3,4	32	4.35	1.68	4.92**	9.03**
1079	Autumn Pakistan Offshore Front	3	32	2.90	1.18	-8.82*	-1.3
1080	Pakistan-India Front	1,2,3,4	32	3.20	1.66	0.08	7.80**
1081	Spring Gulf of Kutch Offshore Front	1	32	2.29	0.98	0.59	1.05
1082	Gujarat Coastal Front	1,2,3,4	32	3.60	1.30	3.66*	5.25**
1083	Gulf of Khambhat Front	3,4	32	3.98	1.51	3.3	3.59**
1084	Gulf of Khambhat Offshore Front	1,4	32	2.95	0.87	-1.92	-2.61*
1085	West India Peninsula Front	1,2,3,4	32	3.46	1.00	2.19*	2.50**
1086	Maldive Islands Discontinuous Front	1,2,3,4	32	1.63	0.40	10.23**	-0.67
1087	Spring North Maldive Islands Front	1		1.71	0.43	13.56**	0
1088	Central Maldive Islands Front	1,4		1.77	0.42	4.33	-3.92*
1089	Huvadhoo Atoll Front	1,3,4		1.66	0.42	9.49**	0.06
1090	South Maldive Islands Front	1,3,4		1.62	0.40	2.49	-2.58
1091	North Chagos Archipelago Front	1,2		2.68	0.55	3.84	-2.46
1092	Summer Chagos Archipelago Front	2		3.92	0.80	2.09	-0.24
1093	Winter Kanyakumari Offshore Front	4	32	3.74	0.91	4.44*	-0.49
1094	Gulf of Mannar Front	1,2,3,4	34	3.95	1.17	2.34	0.55
1095	Palk Strait Front	1,2,3,4	34	3.75	1.14	4.80**	-1.04
1096	East Sri Lanka Front	1,2,3,4	34	3.03	0.82	5.81**	2.42*
1097	East Sri Lanka Offshore Front	2,3	34	2.54	0.64	-4.03	-1.36
1098	East India Peninsula Front	1,2,3,4	34	3.55	0.96	4.75**	3.00**
1099	Winter Ganges Fan Front	4	34	4.83	2.13	7.13**	11.99**
1100	West Ganges-Brahmaputra Estuary Front	1,2,3,4	34	3.58	1.32	-1.39	5.01**
1101	Winter East Ganges-Brahmaputra Estuary Front	4	34	5.23	2.53	0.82	9.51**
1102	East Ganges-Brahmaputra Estuary Front	1,2	34	2.36	0.98	-4.83*	4.84**
1103	West Myanmar Front	1,2,3,4	34	2.95	1.01	-2.42*	1.43
1104	Andaman Islands Front	1,2,4	34	2.48	0.67	4.61*	-1.7
1105	Summer Andaman Islands Front	2	34	2.64	0.48	-2.09	0.33
1106	Spring North Nicobar Islands Front	1	34	2.10	0.65	-2	1.02
1107	Summer South Nicobar Islands Front	2	34	2.81	0.49	-3.17	-1.49
1108	Winter Gulf of Martaban Offshore Front	4	34	4.32	1.26	1.04	0.23
1109	Mouths of the Irrawaddy Coastal Front	1,2,3,4	34	2.77	0.89	4.63**	2.48*
1110	Autumn Mouths of the Irrawaddy Offshore Front	3	34	2.72	0.68	-8.08*	-0.94
1111	East Andaman Sea Front	1,2,3,4	34	3.05	0.80	-3.57**	-0.97
1112	Summer Moscos Islands Offshore Front	2	34	2.33	0.44	2.96	3.54*

1113	Mergui Archipelago Offshore Front	1,4	34	2.46	0.80	8.24**	-1.36
1114	Esat Strait of Malacca Front	1,2,3,4	34	2.96	0.75	-5.81**	-2.24*
1115	South Andaman Sea Front	2,3	34	3.49	0.75	-0.88	2.26
1116	Breueh Island Front	1,2,3,4	34	2.89	0.74	-3.54**	-5.19*
1117	West Strait of Malacca Front	1,2,3	34	2.57	0.61	-0.74	-1.54
1118	Autumn North Strait of Malacca Front	3	34	3.34	0.68	-7.39	0.45
1119	Winter Central Strait of Malacca Front	4	34	3.75	0.89	-4.92	-6.96**
1120	South Strait of Malacca Front	1,4	34	2.74	0.70	-1.22	-2.31
1121	Kauai Island Front	1,2,3,4	10	3.55	0.72	7.54**	-0.51
1122	Oahu Island Front	1,2,3,4	10	4.14	0.83	5.38**	-1.29
1123	Molokai-Maui Island Front	1,2,3,4	10	4.23	0.94	6.92**	0.28
1124	North Island of Hawaii Front	1,2,3,4	10	2.81	0.81	7.62**	-0.34
1125	South Island of Hawaii Front	1,2,3,4	10	2.58	0.71	9.84**	1.61
1126	Gardner Pinnacles Front	1,2,3,4	10	2.86	0.72	1.94	-2.33
1127	Winter French Frigate Shoals Front	4	10	3.17	0.78	-1.92	-6.83*
1128	Hawaii Necker Island Front	1,2	10	2.87	0.66	8.44*	0.96
1129	Summer Ross Ice Shelf Edge Front	4	61	4.00	2.46	-0.53	0.7
1130	Summer Terra Nova Bay Front	4	61	3.16	0.86	3.53	4.85*
1131	Summer West Ross Sea Front	4	61	4.04	0.74	3.17	3.9
1132	Summer West Pennell Bank Front	4	61	3.38	0.51	5.51*	6.15*
1133	Summer North Ross Sea Front	4	61	3.17	0.45	-1.73	-0.22
1134	Summer North Ross Island Front	4	61	2.23	0.53	13.28	19.53*
1135	Summer Ross Bank Front	4	61	2.88	0.43	-6.1	-2.57
1136	Summer East Ross Sea Front	4	61	2.82	0.48	3.88	3.48
1137	Summer West Marie Byrd Land Front	4	61	1.00	0.17	3.96*	11.46*
1138	Summer East Marie Byrd Land Front	4	61	4.36	0.52	4.78	13.61**
1139	Summer West Amundsen Sea Front	4	61	4.56	0.81	4.88*	8.96**
1140	Summer East Amundsen Sea Front	4	61	2.88	0.52	18.28**	25.96**
1141	Summer Pine Islabd Bay Front	4	61	3.33	0.69	14.16**	18.87**
1142	Summer Smyley Island Front	4	61	4.03	0.61	0.65	14.23**
1143	Summer South Alexander Island Front	4	61	2.49	0.66	22.16**	29.21**
1144	Summer South Rothschild Island Front	4	61	4.38	0.96	6.04*	18.43**
1145	Charcot Island Front	1,4	61	2.48	0.54	0	4.81
1146	Autumn Bellingshausen Sea Front	1	61	2.76	0.33	6.62	10
1147	Marguerite Bay Front	1,4	61	4.72	1.02	0.17	3.91
1148	West Bellingshausen Sea Slope Front	1,4		4.44	0.71	-4.34*	-2.54
1149	Bellingshausen Sea Slope Front	1,4	61	4.29	0.69	-1.46	-2.88
1150	North Bellingshausen Sea Slope Front	1,4		4.07	0.71	-6.69**	-5.39
1151	Bransfield Strait Front	1,2,3,4	61	3.37	0.74	3.46*	4.73**
1152	North South Shetland Islands Front	1,4	61	3.28	0.75	-5.07	-4.04
1153	North South Shetland Islands Offshore Front	1,2,3,4		4.10	0.68	-6.48**	3.67
1154	West South Shetland Islands Offshore Front	1,2,3,4		3.05	0.57	-10.64**	-1.93
1155	West Palmer Archipelago Offshore Front	1,2,4		3.69	0.65	-7.58*	0.84
1156	North Bransfield Strait Front	1,2,3	61	3.63	0.65	4.74*	3.33
1157	South Scotia Ridge Front	1,2,3,4	61	5.47	0.91	-1.88	6.05**

1158	Autumn South Orkney Microcontinent Front	1		3.60	0.56	-7.19*	0.35
1159	North Weddell Sea Front	1,4		3.03	0.49	-2.93	2.56
1160	Joinville Island Group Front	1,4	61	2.05	0.34	-3.34	-2.81
1161	Summer James Ross Island Group Front	4	61	3.72	0.55	-5.81	1.14
1162	Summer Berkner Island Front	4	61	1.00	0.14	0	-0.01
1163	Summer Coats Land Front	4	61	1.90	0.41	-5.41	-1.47
1164	Summer Brunt Ice Shelf Edge Front	4	61	2.73	0.46	2.44	1.85
1165	Summer Brunt Ice Shelf Offshore Front	4		2.16	0.34	-0.28	-3.97
1166	Summer West Queen Maud Land Front	4	61	3.00	0.52	-10.89*	-8.41
1167	West Queen Maud Land Offshore Front	1,4		2.56	0.39	0.99	-0.8
1168	Lazarev Sea Front	1,4	61	3.04	0.52	-2.68	-6.02
1169	Astrid Ridge Front	1,4		3.20	0.44	7.81**	9.13**
1170	North Astrid Ridge Front	1,4		2.52	0.36	-0.23	-1.96
1171	Autumn West Astrid Ridge Front	1		2.24	0.32	-7.27	-10.79*
1172	Autumn East Astrid Ridge Front	1		3.25	0.43	-1.06	-0.59
1173	Autumn West Riiser-Larsen Sea Front	1		2.73	0.34	6.37	4.71
1174	East Riiser-Larsen Sea Front	1,4		2.81	0.44	1.87	3.33
1175	Cosmonauts Sea Front	1,4	61	3.48	0.64	-3.12	-3.5
1176	South Gunnerus Ridge Front	1,4	61	3.27	0.58	6.54*	5
1177	North Gunnerus Ridge Front	1,4		3.03	0.42	0.66	1.68
1178	Kainan Maru Seamount Front	1,4		3.27	0.43	3.52	1.03
1179	Cosmonauts Sea Offshore Front	1,4		4.24	0.66	-0.42	3.02*
1180	Summer Cosmonauts Sea Offshore Front	4		3.94	0.99	2.84	1.04
1181	West Prydz Bay Front	1,4	61	2.56	0.49	-0.99	2.47
1182	Summer Prydz Bay Front	4	61	4.09	1.31	7.37**	8.14*
1183	Summer East Prydz Bay Front	4	61	4.68	1.04	6.41*	8.50**
1184	Prydz Bay Offshore Front	1,4		3.85	0.79	-3.9	4.15
1185	Davis Sea Front	1,4	61	2.75	0.50	-2.99	2.75
1186	West Summer Shackleton Ice Shelf Front	4	61	5.12	0.91	-3.70*	8.07**
1187	Shackleton Ice Shelf Offshore Front	1,4		5.22	0.95	-3.35*	6.34**
1188	Davis-Mawson Sea Front	1,4	61	4.06	0.92	-6.68**	-3.53
1189	Summer Vincennes Bay Front	4	61	5.22	0.77	0.14	5.41*
1190	Summer Moscow University Ice Shelf Front	4	61	3.79	0.58	1.58	3.78
1191	Mawson-DUrville Sea Offshore Front	1,4		5.98	1.02	-3.51*	2.31*
1192	DUrville-Somov Sea Offshore Front	1,4		5.32	1.09	-1.68	3.38*
1193	West Adélie Basin Front	1,4	61	4.38	1.04	-1.4	1.67
1194	Summer South Adélie Basin Front	4	61	3.64	0.64	-3.18	0.14
1195	Summer North Adélie Bank Front	4		4.94	1.14	1.45	5.39
1196	Summer Ninnis Bank Front	4	61	4.85	0.93	-2.43	-5.68**
1197	Summer North Balleny Islands Front	4		2.99	0.47	-5.67	-4.6
1198	Summer South Balleny Islands Front	4		2.63	0.39	-7.15	-11.58

Supplementary References

1. Belkin, I. M., Cornillon, P. C. & Sherman, K. Fronts in Large Marine Ecosystems. *Prog.*

Oceanogr. **81**, 223-236 (2009).
