# Supplementary material for: Global mapping and evolution of persistent fronts in Large Marine Ecosystems over the past 40 years
Source: Nat Commun. 2024 May 14;15:4090. doi: 10.1038/s41467-024-48566-w (PMC11094120; doi:10.1038/s41467-024-48566-w)
Supplement: Supplementary file 3 — Reporting Summary [file 41467_2024_48566_MOESM3_ESM.pdf]

nature portfolio

Corresponding author(s): Haiqing Yu

Last updated by author(s): Apr 28, 2024

Reporting Summary

Nature Portfolio wishes to improve the reproducibility of the work that we publish. This form provides structure for consistency and transparency in reporting. For further information on Nature Portfolio policies, see our Editorial Policies and the Editorial Policy Checklist.

Statistics

For all statistical analyses, confirm that the following items are present in the figure legend, table legend, main text, or Methods section.

n/a	Confirmed	
x		The exact sample size (*n*) for each experimental group/condition, given as a discrete number and unit of measurement
x		A statement on whether measurements were taken from distinct samples or whether the same sample was measured repeatedly
	x	The statistical test(s) used AND whether they are one- or two-sided<br>*Only common tests should be described solely by name; describe more complex techniques in the Methods section.*
x		A description of all covariates tested
x		A description of any assumptions or corrections, such as tests of normality and adjustment for multiple comparisons
	x	A full description of the statistical parameters including central tendency (e.g. means) or other basic estimates (e.g. regression coefficient) AND variation (e.g. standard deviation) or associated estimates of uncertainty (e.g. confidence intervals)
x		For null hypothesis testing, the test statistic (e.g. *F*, *t*, *r*) with confidence intervals, effect sizes, degrees of freedom and *P* value noted<br>*Give P values as exact values whenever suitable.*
x		For Bayesian analysis, information on the choice of priors and Markov chain Monte Carlo settings
x		For hierarchical and complex designs, identification of the appropriate level for tests and full reporting of outcomes
x		Estimates of effect sizes (e.g. Cohen's *d*, Pearson's *r*), indicating how they were calculated

Our web collection on statistics for biologists contains articles on many of the points above.

Software and code

Policy information about availability of computer code

Data collection	The satellite-observed SST dataset is available from https://doi.org/10.48670/moi-00169, the reanalysis SST dataset is accessible from https://doi.org/10.48670/moi-00021, the Earth system model datasets of HadGEM3 and FGOALS can be accessed from https://esgf-node.llnl.gov/projects/cmip6/. The identified persistent fronts in this work is available from https://doi.org/10.5281/zenodo.10210578, and source data are provided with this paper.
Data analysis	The improved front detection algorithm is available from https://doi.org/10.1016/j.rse.2023.113627, the detection algorithm of persistent frontal pixels can be accessed from https://doi.org/10.5281/zenodo.1021057881, the code of Mann-Kendall test and Sen's slope estimator is accessible from https://www.mathworks.com/matlabcentral/fileexchange/22389-seasonal-kendall-test-with-slope-for-serial-dependent-data.

For manuscripts utilizing custom algorithms or software that are central to the research but not yet described in published literature, software must be made available to editors and reviewers. We strongly encourage code deposition in a community repository (e.g. GitHub). See the Nature Portfolio guidelines for submitting code & software for further information.

Data

Policy information about availability of data

All manuscripts must include a data availability statement. This statement should provide the following information, where applicable:

- Accession codes, unique identifiers, or web links for publicly available datasets
- A description of any restrictions on data availability
- For clinical datasets or third party data, please ensure that the statement adheres to our policy

The satellite-observed SST dataset is available from https://doi.org/10.48670/moi-00169, the reanalysis SST dataset is accessible from https://doi.org/10.48670/moi-00021, the Earth system model datasets of HadGEM3 and FGOALS can be accessed from https://esgf-node.llnl.gov/projects/cmip6/. The identified persistent fronts in this work is available from https://doi.org/10.5281/zenodo.10210578, and source data are provided with this paper.

Research involving human participants, their data, or biological material

Policy information about studies with human participants or human data. See also policy information about sex, gender (identity/presentation), and sexual orientation and race, ethnicity and racism.

Reporting on sex and gender	not applicable
Reporting on race, ethnicity, or other socially relevant groupings	not applicable
Population characteristics	not applicable
Recruitment	not applicable
Ethics oversight	not applicable

Note that full information on the approval of the study protocol must also be provided in the manuscript.

Field-specific reporting

Please select the one below that is the best fit for your research. If you are not sure, read the appropriate sections before making your selection.

☐ Life sciences ☐ Behavioural & social sciences ☒ Ecological, evolutionary & environmental sciences

For a reference copy of the document with all sections, see nature.com/documents/nr-reporting-summary-flat.pdf

Ecological, evolutionary & environmental sciences study design

All studies must disclose on these points even when the disclosure is negative.

Study description	We adopted global daily satellite-observed SST data to study ocean fronts.
Research sample	not applicable
Sampling strategy	not applicable
Data collection	We opted to employ a global gap-free SST reprocessed product, made available by the Copernicus Marine Environment Monitoring Service (CMEMS, https://doi.org/10.48670/moi-00169), for the identification of persistent fronts and their evolution. This dataset was developed and processed by ESA SST CCI and C3S, using the Operational SST and Sea Ice Analysis (OSTIA) derived from multiple reprocessed satellite measurements.
Timing and spatial scale	We adopted global daily satellite-observed SST data from 1982 to 2021.
Data exclusions	No data were excluded from analyses.
Reproducibility	No experiments were designed and conducted in this study.
Randomization	not applicable
Blinding	not applicable

Did the study involve field work? ☐ Yes ☒ No

Reporting for specific materials, systems and methods

We require information from authors about some types of materials, experimental systems and methods used in many studies. Here, indicate whether each material, system or method listed is relevant to your study. If you are not sure if a list item applies to your research, read the appropriate section before selecting a response.

Materials & experimental systems

n/a	Involved in the study
☒	☐ Antibodies
☒	☐ Eukaryotic cell lines
☒	☐ Palaeontology and archaeology
☒	☐ Animals and other organisms
☒	☐ Clinical data
☒	☐ Dual use research of concern
☒	☐ Plants

Methods

n/a	Involved in the study
☒	☐ ChIP-seq
☒	☐ Flow cytometry
☒	☐ MRI-based neuroimaging

Plants

Seed stocks	not applicable
Novel plant genotypes	not applicable
Authentication	not applicable
